# Repurposing the Antidepressant Sertraline: A Systematic Scoping Review of Its Anticancer Mechanisms

**DOI:** 10.1002/prp2.70168

**Published:** 2025-08-28

**Authors:** Ciara B. Blum, McCarlie‐Jayne Dohrmann, Lucia McCarthy, Milli McMenamin, Liam A. O'Callaghan

**Affiliations:** ^1^ School of Medicine and Dentistry Griffith University Southport Queensland Australia; ^2^ Faculty of Health Sciences and Medicine Bond University Robina Queensland Australia

**Keywords:** antineoplastic agents, apoptosis, drug repositioning, drug resistance, sertraline

## Abstract

Drug repurposing offers a cost‐effective and time‐efficient strategy for identifying new cancer therapies. Sertraline, a widely prescribed selective serotonin reuptake inhibitor (SSRI), has shown promising anticancer properties through modulation of key pathways involved in tumor survival, stress adaptation, and therapeutic resistance. This scoping review systematically evaluates the current evidence on sertraline's anticancer mechanisms, efficacy, and translational potential. A systematic search of PubMed, EMBASE, Scopus, and Web of Science was conducted in accordance with PRISMA‐ScR guidelines. Eligible studies included in vitro, in vivo, and clinical investigations. Data on cancer types, mechanisms, assays, and outcomes were extracted and synthesized. Of 97 screened articles, 67 met inclusion criteria, comprising 56 preclinical studies, nine population‐based studies, and two mixed‐methods reports. Sertraline induces apoptosis via mitochondrial dysfunction, caspase activation, and Bcl‐2 downregulation, disrupts autophagy and the unfolded protein response, and impairs serine/glycine metabolism through SHMT inhibition. It also suppresses oncogenic signaling via mTOR and TCTP modulation. In vivo studies confirmed tumor growth inhibition in various cancer models, including breast, lung, glioblastoma, and liver. Sertraline enhances the efficacy of chemotherapy, radiotherapy, and targeted therapies by sensitizing resistant cells, modulating immune responses, and impairing metabolic recovery. Retrospective studies suggest no increased cancer risk with SSRI use and hint at protective associations in select malignancies. While current evidence is predominantly preclinical, sertraline's multi‐targeted action and established safety profile support its candidacy for repurposing. Further translational research and biomarker‐driven clinical trials are warranted to validate its therapeutic niche and optimize its integration into oncology.

## Graphical abstract

A multi‐targeted antidepressant with strong preclinical anticancer activity, sertraline represents a candidate for therapeutic repurposing.

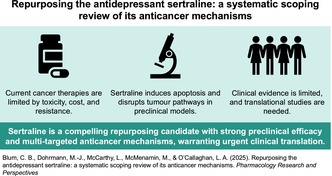

**Abbreviations**
5‐FU: fluorouracil
AMPK: AMP‐activated protein kinase
BAK: Bcl‐2 antagonist/killer 1
BAX: Bcl‐2‐associated X protein
Bcl‐2: B‐cell lymphoma 2
Bcl‐xL: B‐cell lymphoma‐extra large
CDC7: cell division cycle 7‐related protein kinase
CHOP: C/EBP homologous protein
Mcl‐1: myeloid cell leukemia sequence 1
MRP1/7: multidrug resistance‐associated protein 1/7
mTOR: mechanistic target of rapamycin
PERK: protein kinase RNA‐like endoplasmic reticulum kinase
SHMT: serine hydroxymethyltransferase
SOX2: SRY‐box transcription factor 2
STAT3: signal transducer and activator of transcription 3
TCTP: translationally controlled tumor protein
TP53: tumor protein p53

## 1 Introduction

Cancer represents a major global health burden, with approximately 20 million new cases and 10 million deaths recorded globally in 2022 [1]. Partly due to factors such as population growth and aging, this burden is expected to rise substantially, with projections estimating 35.3 million new cases by the year 2050—to an increase of nearly 77% from 2022 [2]. While substantial progress has been made in the development of anticancer drugs across the years, this forecast highlights a pressing need for the establishment of more effective treatment modalities. However, it can take approximately 13–15 years and cost around US $2–3 billion to bring a new drug to the market [3]. This lengthy and expensive process can be mitigated by repurposing existing drugs, which have pre‐established pharmacological and safety profiles. Repurposed drugs can often enter the development timeline in its clinical trial (third) phase, accelerating this process by up to 7 years and costing a substantially lower average of US $300 million [3]. Other benefits of drug repurposing include improved return on investment and a decreased likelihood of the drug failing in subsequent efficacy trials, especially from a safety perspective [4].

In recent years, the idea of repurposing drugs to treat common and rare conditions, including cancer, has garnered attention in the literature. One such candidate is sertraline, known by its brand name Zoloft, a selective serotonin reuptake inhibitor (SSRI) marketed for the treatment of depression, anxiety, and other mood disorders [5]. Sertraline is a naphthylamine derivative with a key structural feature of a 3,4‐dichlorophenyl group [6]. It primarily inhibits serotonin (5‐HT) reuptake, leading to increased serotonin levels in synaptic clefts and enhanced serotonergic neurotransmission, while having minimal effect on dopamine and noradrenaline reuptake [6]. The anticancer potential of sertraline was first recognized in 1993 by French researchers Adam Telerman and Robert Amson. Telerman et al. [7] also discovered that TCTP, which was subsequently found to secrete histamine, is a key player in tumor regression. This inspired research into whether antihistamines and chemically related drugs, including antipsychotics and antidepressants, could interfere with the activity of TCTP to target cancer.

The aim of this scoping review is to systematically map and synthesize the existing literature on the anticancer potential of sertraline, focusing on its preclinical and clinical efficacy, cancer type, and current gaps in knowledge. Guided by these objectives, this review seeks to establish a basis not only for understanding the potential of sertraline as a repurposed cancer therapy, but also for informing future investigations into its clinical utility. While there is early evidence to suggest that sertraline has potential in the field of oncology, further research is crucial to clarify its mechanisms of action and its suitability as either a therapeutic adjunct or standalone treatment modality. Building on previous work examining the anticancer effects of psychotropic drugs, including aripiprazole [8], this review explores the potential role of sertraline in cancer treatment. The following research questions were developed to systematically assess the existing evidence on sertraline's anticancer properties:

1. What are the biological mechanisms underlying sertraline's proposed anticancer activity in preclinical models?
2. Which cancer types have been investigated in relation to sertraline treatment, and are there any patterns of sensitivity or resistance that have been observed?
3. How does sertraline affect tumor progression, metastasis, and survival outcomes in animal models?
4. Are there any known interactions between sertraline and standard cancer treatments, such as chemotherapy or radiation therapy?
5. What evidence is available from clinical studies regarding the use of sertraline as an adjunctive or standalone therapy in cancer patients?

## 2 Methodology

### 2.1 Protocol and Registration

This scoping review adheres to the reporting standards outlined by the Joanna Briggs Institute (JBI) and the Preferred Reporting Items for Systematic reviews and Meta‐Analyses extension for Scoping Reviews (PRISMA‐ScR) [9, 10]. An a priori protocol was developed and registered with the Open Science Framework (OSF).

### 2.2 Search Strategy

Following the JBI's three‐step methodological framework, a structured search strategy was developed. An initial literature search was performed on January 3, 2025, using PubMed and Google Scholar to identify relevant keywords and index terms. The primary search terms included “sertraline,” “anticancer,” “apoptosis,” and “cell death.” Additional terms were incorporated to refine and expand the search scope for comprehensive literature retrieval. The finalized search strategies were translated using the Systematic Review Accelerator (SRA) Polyglot [11] (Supporting Information S1). Furthermore, a gray literature search was conducted through Research Rabbit [12], Litmaps [13], and SRA TERA Farmer [14] to ensure a thorough exploration of relevant sources.

### 2.3 Information Sources

The final search of four databases (PubMed, EMBASE, SCOPUS and Web of Science) was conducted on June 5, 2025. The retrieved results were subsequently exported into Endnote X9 [15] for further analysis and organization.

### 2.4 Study Selection

Deduplication was conducted using automation software (SRA Deduplicator) before undergoing manual verification [16]. The remaining articles were then screened by title and abstract within the SRA Screenatron program by two authors (L.A.O. and C.B.B.) [16]. Studies were included if they investigated sertraline's anticancer effects in various contexts, including in vitro, in vivo, or clinical research. Eligible studies had to present original data demonstrating sertraline's impact on cancer‐related outcomes, such as cell viability, apoptosis, or tumor regression. Exclusion criteria encompassed non‐English publications, studies unrelated to therapeutic oncology, articles without original data (e.g., reviews, editorials), and conference abstracts. Full‐text screening was performed in Covidence [17] by two authors (L.A.O. and C.B.B.), with any disagreements resolved through discussion with other members of the research team.

### 2.5 Charting, Collating and Summarizing the Data

Four reviewers (C.B.B., M.‐J.D., L.M., and M.M.) independently extracted data using a predefined data extraction table in Microsoft Excel, following established methodological guidelines [18]. The extraction table included key details such as author, year of publication, country of study, research objectives, sample characteristics (e.g., cancer type, cell lines, and animal models), assays conducted, IC_50_ values, and/or sertraline dosages used in animal studies, as well as key findings. Given that the primary aim of this scoping review is to provide a broad synthesis of the available literature, formal assessments of methodological quality were not conducted [9]. Instead, the emphasis was placed on capturing a diverse range of evidence to highlight research gaps and emerging patterns.

## 3 Results

### 3.1 Search Results

**FIGURE 1:**
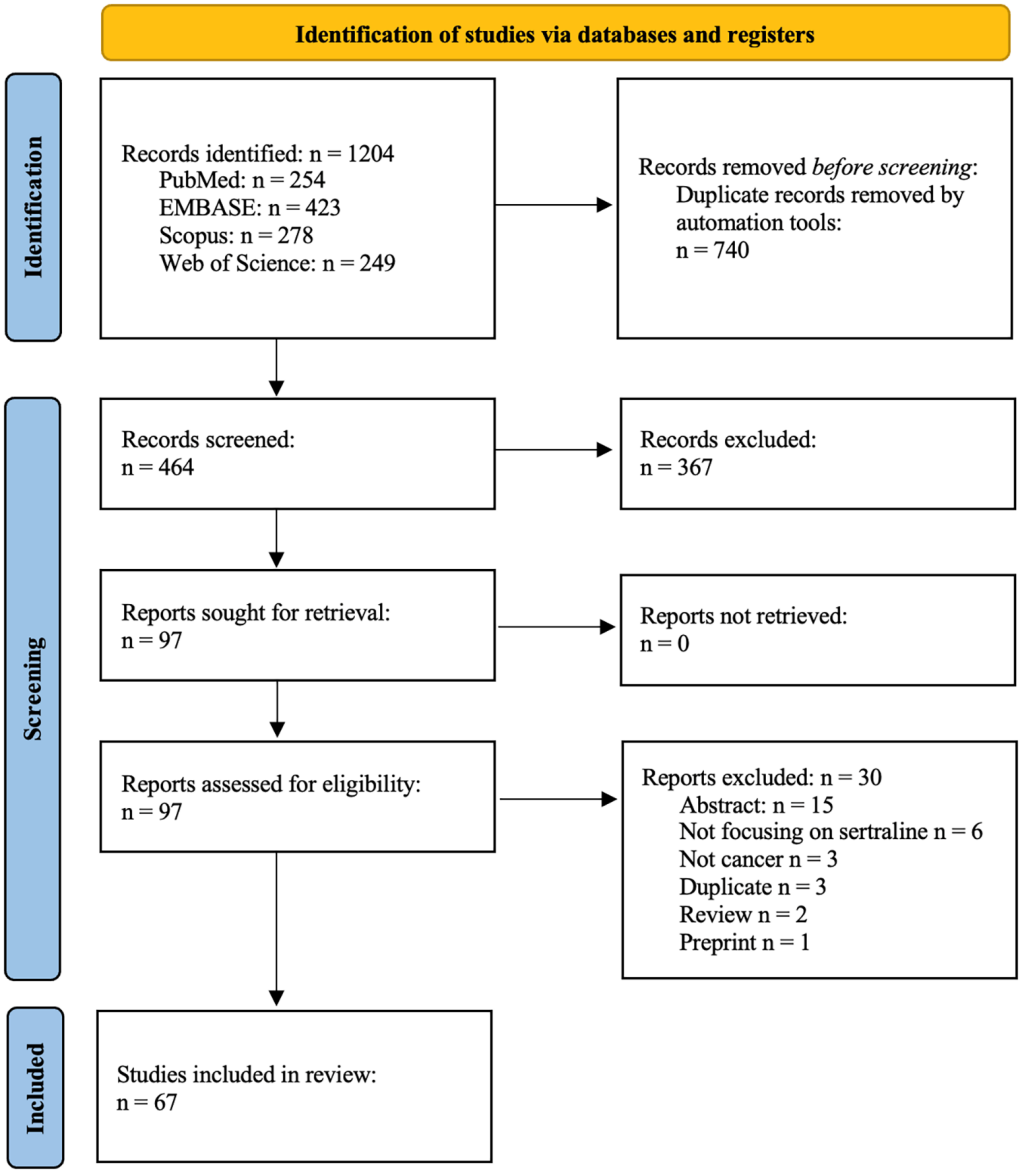
PRISMA‐ScR flow diagram.

Database searching yielded 1204 citations, of which 740 were automatically removed as duplicates. Title and abstract screening of the remaining 464 citations led to the exclusion of 367, leaving 97 articles for full‐text screening. After retrieving and screening these 97 articles, 30 articles were excluded, leaving a final number of 67 articles from the primary database search (Figure 1).

### 3.2 Synthesis of Results

A total of 67 unique studies met the inclusion criteria for this review. Of these, 56 reported laboratory‐based in vitro or in vivo investigations (Table 1), 9 were population‐level studies (Table 2), and 2 integrated both laboratory and epidemiological data. To facilitate interpretation, the dual‐design studies are also summarized separately (Table 3). Across all study types, sertraline's anticancer effects were explored in a diverse range of cancers, with breast, colorectal, brain, liver, and lung cancers receiving the most attention (Table 4). The in vitro and in vivo studies encompassed a broad international cohort of research settings, reflecting widespread scientific interest in sertraline as a potential anticancer agent. Overall, these studies collectively support a multifaceted anticancer profile for sertraline, spanning direct cytotoxicity in tumor models to possible protective associations in population‐based data.

**TABLE 1:** Summary of in vitro and in vivo studies investigating the anticancer properties of sertraline, organized by year of publication.

Author/year/country	Research aims	Sample details	Assays performed	IC_50_ value(s)/animal model	Key findings
Tuynder et al. (2004), France [19]	Investigate TCTP as a key target in tumor reversion Assess whether TCTP downregulation can induce tumor reversion in various solid cancers (colon, lung, melanoma) Identify pharmacological compounds, including sertraline, that suppress TCTP and promote cancer cell death	*Cancer type*: Colon Lung Skin Breast Leukemia *Cell lines used*: DLD‐1 A549 WM‐266‐4 WM‐115 SK‐MEL‐28 Hs852T MDA‐MB231 U937 *Animal model*: SCID/SCID mice injected with 10^7^ tumor cells subcutaneously into the right flank	Soft agar assay Western blotting PCR IF ATP‐based cell viability assay Flat reversion assay RNA interference Drug screening for cytotoxicity and TCTP suppression Tumor volume measurement in SCID/SCID mice Drug treatment response assessment	*IC*_*50*_: Not reported *Animal model*: Subcutaneous tumors from MDA‐MB231 and U937 cells were induced by injecting 10^7^ cells into the right flank of SCID/SCID mice Treatment (promethazine at 22.5 mg/kg, sertraline at 18.0 mg/kg, and thioridazine at 6.75 mg/kg) started: ○ Early‐stage: 2 days before tumor cell injection ○ Late‐stage: when tumors reached 4 mm^3^	TCTP is significantly downregulated during tumor reversion Inhibition of TCTP reduces malignancy in multiple solid tumors Sertraline and other neuroleptic/antihistaminic compounds lower TCTP levels and induce cancer cell death In vivo, sertraline suppresses tumor growth in SCID mice without causing toxicity Sertraline's anticancer effects are linked to TCTP inhibition, supporting its repurposing potential TCTP silencing induces flat revertants in transformed fibroblasts, confirming its role in malignancy reversal
Reddy et al. (2006), United States of America [20]	To investigate the cytotoxic effects of sertraline on melanoma cells To determine whether sertraline inhibits the Akt signaling pathway, which plays a role in melanoma resistance to treatment To assess whether sertraline reduces melanoma tumor growth in vivo	*Cancer type*: Skin (melanoma) *Cell lines used*: A375 B16 *Animal model*: 6‐week‐old nude male mice injected with A375 cells	Cell viability assays Western blotting Flow cytometry Caspase activity assay In vivo tumor growth assay (xenograft model)	*IC*_*50*_: Not reported *Animal model*: A375 melanoma‐bearing nude mice (3 weeks post‐inoculation) received: ○ Sertraline: 1 mg/day (IP.) in 20% Intralipid (0.3 mL) ○ Vehicle control: 20% Intralipid (0.3 mL, IP)	Sertraline significantly inhibited melanoma cell proliferation in a dose‐dependent manner It downregulated the Akt signaling pathway Sertraline induced ER stress, leading to apoptosis in melanoma cells In vivo, sertraline monotherapy modestly reduced melanoma tumor growth, but the effect was not statistically significant The study suggests that sertraline may enhance the efficacy of existing melanoma treatments by inhibiting Akt and inducing ER stress
Gil‐Ad et al. (2008), Israel [21]	Assess sertraline's effects on colorectal cancer cell viability and proliferation Examine its influence on cell cycle, apoptosis, and key molecular pathway Compare cytotoxicity with doxorubicin, vincristine, and 5‐FU	*Cancer type*: Colorectal *Cell lines used*: HT29 LS1034 *Animal model*: CD1 nude mice xenografted subcutaneously with HT29 cells	Cell viability assay (neutral red staining)[3H]‐thymidine incorporation assay Flow cytometry Fluorescence microscopy Caspase‐3 activity Western blot In vivo tumor growth study	*IC*_*50*_: HT29 = 14.7 μM LS1034 = 13.1 μM *Animal model*: Sertraline (15 mg/kg IP, 3×/week) significantly inhibited tumor growth in HT29 xenografted mice	Sertraline demonstrated strong anticancer activity in colorectal cancer, particularly in multi‐drug‐resistant cells, through a combination of cell cycle arrest, apoptosis induction, Bcl‐2 inhibition, and c‐Jun activation Sertraline showed higher efficacy in multi‐drug resistant LS1034 cells than traditional chemotherapy drugs
Amit et al. (2009), Israel [22]	To evaluate the cytotoxic effects of SSRIs on malignant T cells (Jurkat) To assess the ability of SSRIs to act as chemosensitizers in combination with conventional chemotherapy	*Cancer type*: T‐cell lymphoma/leukemia *Cell lines used*: Jurkat human T‐cell leukemia cells *Animal model*: None	Alamar Blue viability [3H] thymidine incorporation Caspase‐3 enzymatic activity Fluorescence microscopy Western blot analysis (Bcl‐2, MAPK pathway proteins)	*IC*_*50*_: Sertraline = 9.5 μM Combination treatments: ○ Sertraline (7.5 μM) + doxorubicin reduced doxorubicin IC_50_ from 0.075 to 0.025 μM ○ Sertraline (7.5 μM) + vincristine reduced vincristine IC_50_ from 32.5 to 12.9 μM	Sertraline reduced Jurkat cell viability Sertraline induced apoptosis via caspase‐3 activation and reduced Bcl‐2 expression No activation of MAPK pathway proteins Enhanced chemotherapy effects of doxorubicin and vincristine (lowered IC_50_) Potential chemosensitizer; further in vivo studies needed
Lin et al. (2010), Canada [23]	Investigate the anticancer effects of sertraline Examine its role in inhibiting translation initiation and mTOR signaling in cancer cells Assess whether sertraline enhances chemosensitivity to doxorubicin in an Eμ‐Myc murine lymphoma model	*Cancer type*: Breast Lymphoma *Cell lines used*: MCF‐7 TSC2^+/+^p53^−/−^ TSC2^−/−^p53^−/−^ *Animal model*: 6–8‐week‐old female C57BL/6 mice, injected with 2 × 10^6^ Pten+/−Eμ‐Myc, Eμ‐Myc/Bcl‐2, or Eμ‐Myc/eIF4E lymphoma cells	Sulforhodamine B assay Protein synthesis assay Polysome profile analysis Western blot RNA interference IF microscopy Flow cytometry TUNEL assay IHC Animal survival analysis	*IC*_*50*_: MCF‐7 = 25 μM (24‐h treatment) *Animal model*: Sertraline: 20 mg/kg daily for 5 days (IP) Rapamycin: 4 mg/kg daily for 5 days (IP) Doxorubicin: 10 mg/kg (single dose, IP) Combination treatments: ○ Sertraline (20 mg/kg, daily for 5 days) + doxorubicin (10 mg/kg, single dose on day 2) ○ Rapamycin (4 mg/kg, daily for 5 days) + doxorubicin (10 mg/kg, single dose on day 2)	Sertraline inhibited cell proliferation in MCF‐7 cells via inhibition of translation initiation, downregulation of eIF4F complex and increased eIF2α phosphorylation and REDD1 expression, leading to suppression of mTOR signaling Sertraline sensitized Pten^+/−^Eμ‐Myc tumors to doxorubicin, increasing tumor‐free survival compared to monotherapy No synergistic effect was seen in Eμ‐Myc/Bcl‐2 or Eμ‐Myc/eIF4E tumors, suggesting the response is genotype‐dependent In vivo, sertraline treatment reduced protein synthesis, as shown by polysome profile analysis in tumors
Tzadok et al. (2010), Israel [24]	To investigate the antiproliferative effects of combining psychotropic drugs with conventional glioblastoma treatments and the tyrosine kinase inhibitor imatinib in U87 glioblastoma cells	*Cancer type*: Glioblastoma *Cell lines used*: U87 *Animal model*: None	SRB assay Flow cytometry ELISA Caspase‐3 activity assay Western blotting (AKT/pAKT and MAPK/pMAPK) ATP content measurement	*IC*_*50*_: Not reported *LD*_*50*_: 8 μM	Sertraline (8 μM) reduced U87 glioblastoma cell proliferation in a dose‐dependent manner In combination with imatinib (10 μM), it produced a synergistic antiproliferative effect This combination significantly reduced pAKT expression (~70%), suggesting disruption of survival signaling No significant changes in pMAPK, caspase‐3 activity, or ATP beyond imatinib alone Minor G2/M cell cycle arrest, but not enough to explain cell death Mechanism likely non‐apoptotic, possibly involving mitochondrial dysfunction
Chien et al. (2011), Taiwan [25]	To investigate whether sertraline alters cytosolic free Ca^2+^ levels ([Ca^2+^]i) in OC2 human oral cancer cells To determine the mechanisms underlying Ca^2+^ influx and release	*Cancer type*: Oral *Cell lines used*: OC2 *Animal model*: None	Fura‐2 fluorescence Ca^2+^ imaging Mn^2+^ quenching assay Pharmacological inhibition experiments	*IC*_*50*_: Not reported	Sertraline induced a concentration‐dependent rise in [Ca^2+^]i in OC2 oral cancer cells Ca^2+^ release was mediated via ER stores and store‐operated Ca^2+^ entry channels Inhibited by phospholipase A2 inhibitors but not by phospholipase C inhibitors Store‐operated Ca^2+^ channel blockers (nifedipine, econazole, SKF96365) reduced sertraline‐induced Ca^2+^ influx
Huang et al. (2011), Taiwan [26]	Investigate whether sertraline alters basal [Ca2+]i levels in suspended PC3 human prostate cancer cells by using fura‐2 as a Ca^2+^‐sensitive fluorescent probe Investigate the effect of sertraline on cell viability and apoptosis	*Cancer type*: Prostate *Cell lines used*: PC3 *Animal model*: None	[Ca^2+^]i measurements WST‐1 MTT Flow cytometry Mn^2+^ quenching assay Store depletion assay Inhibitor studies	*IC*_*50*_: Not reported	In PC3 prostate cancer cells, sertraline induced [Ca^2+^]i rise via phospholipase C‐dependent ER release and store‐operated Ca^2+^ influx pathways Apoptosis occurred independently of [Ca^2+^]i rise
Lin et al. (2013), Taiwan [27]	Investigate whether sertraline affects intracellular calcium levels ([Ca^2+^]i) in MG63 human osteosarcoma cells Assess whether sertraline induces cytotoxicity and apoptosis in MG63 cells Determine the mechanisms involved in Ca^2+^ influx and release in response to sertraline	*Cancer type*: Osteosarcoma *Cell lines used*: MG63 *Animal model*: None	Intracellular calcium ([Ca^2+^]i) measurement u MTT assay Flow cytometry DCFH‐DA Pharmacological inhibition assays (blockers of Ca^2+^ entry pathways, ER Ca^2+^ release, and phospholipase C activity)	*IC*_*50*_: Not reported	Sertraline induced a [Ca^2+^]i rise in MG63 cells in a concentration‐dependent manner (50–200 μM) Ca^2+^ influx was mediated by L‐type Ca^2+^ channels and store‐operated Ca^2+^ entry (SOCE) ER Ca^2+^ release was PLC‐dependent, as it is blocked by the PLC inhibitor U73122 Sertraline decreased MG63 cell viability at 20–30 μM after 24‐h treatment Sertraline‐induced cytotoxicity was independent of Ca^2+^, as chelating intracellular Ca^2+^ did not reverse cell death Apoptosis was triggered at 30 μM Sertraline increased ROS production at 20 and 30 μM, suggesting that oxidative stress contributes to apoptosis
Chen et al. (2014), United States of America [28]	Investigate sertraline‐induced molecular mechanisms of hepatic toxicity in HepG2 cells Identify and characterize ER stress as a novel mechanism Examine the involvement of ER stress and MAPK signaling pathway Assess the link between ER stress, apoptosis, and MAP4K4‐JNK pathway regulation	*Cancer type*: Liver *Cell lines used*: HepG2 *Animal model*: None	Whole genome gene expression microarray analysis qPCR Western blotting X‐box binding protein 1 (XBP1) mRNA splicing assay Gaussian luciferase secretion Secreted alkaline phosphatase secretion Caspase‐3/7 luminescence apoptosis assay Treatment with 4‐phenylbutyrate (ER stress inhibitor) and JNK inhibitor SP600125 MAP4K4 silencing via shRNA	*IC*_*50*_: 25.7 μM (measured by LDH 6 h treatment)	Sertraline induces ER stress in HepG2 liver cancer cells, upregulating key markers (PERK, IRE1α, CHOP, ATF4, phospho‐eIF2α) in a dose‐ and time‐dependent manner ER stress occurs before cytotoxicity, suggesting it is an early response ER stress is linked to apoptosis, as shown by caspase activation and CHOP induction; inhibition with 4‐PBA reduces both ER stress and apoptosis MAP4K4‐JNK signaling regulates sertraline‐induced ER stress and apoptosis; silencing MAP4K4 or using a JNK inhibitor attenuates effects Pathway analysis revealed alterations in ER stress, MAPK, cell cycle, p53, and other cancer‐related pathways Sertraline's cytotoxicity in hepatic cells involves interconnected mitochondrial dysfunction, ER stress, apoptosis, and MAPK pathway activation
Chen et al. (2014), United States of America [29]	Investigate whether sertraline induces cytotoxicity and apoptosis in human hepatoma HepG2 cells Characterize the molecular mechanisms involved, focusing on intrinsic and extrinsic apoptotic pathways	*Cancer type*: Liver *Cell lines used*: HepG2 *Animal model*: None	LDH release assay Cellular ATP measurement Mitochondrial membrane potential (JC‐1) Flow cytometry Caspase‐3/7 and caspase‐9 activity assays Western blotting (caspases, cytochrome c, Bcl‐2 family, phospho‐JNK, ERK, p38, c‐Jun) Real‐time PCR Pharmacological inhibition (caspase inhibitors, JNK/p38/ERK inhibitors) MAP4K4 gene silencing via shRNA	*IC*_*50*_: Not reported	Sertraline reduced HepG2 viability and ATP levels in a dose‐ and time‐dependent manner (6.25–50 μM) Caused mitochondrial membrane depolarization and cytochrome c release Activated caspase‐9 and caspase‐3/7, indicating apoptosis via intrinsic pathway Increased TNF expression (~66‐fold at 50 μM) and activated JNK, ERK, and p38 JNK inhibition (SP600125) and MAP4K4 silencing significantly reduced apoptosis and cell damage Reduced expression of anti‐apoptotic Bcl‐2 and Mcl‐1 proteins; pro‐apoptotic proteins largely unchanged Apoptosis driven primarily via TNF–MAP4K4–JNK signaling, with both intrinsic and extrinsic caspase pathways involved
Drinberg et al. (2014), Israel [30]	Investigate whether sertraline can overcome multidrug resistance in cancer by inhibiting P‐gp drug efflux pumps Test the efficacy of sertraline with Doxil (pegylated liposomal doxorubicin) to enhance chemotherapy in drug‐resistant tumors	*Cancer type*: Ovarian *Cell lines used*: NCI‐ADR/RES (NAR) OVCAR‐8 *Animal model*: 6‐week‐old athymic nude mice, subcutaneously injected with 4 × 10^6^ NCI‐ADR/RES (NAR) cells	Pump expression analysis (flow cytometry) Pump functionality (efflux assays) XTT assay Drug accumulation and efflux assay (fluorescence‐based) Tumor volume measurement Survival analysis (Kaplan–Meier method) Preparation and characterization of pegylated liposomal doxorubicin (Doxil)	*IC*_*50*_: Not reported *Animal model*: Doxorubicin experiment: ○ Saline and DOX (2 mg/kg) administered IV every 3 days for a total of 12 injections DOXIL Experiment: ○ Saline and DOXIL (2 mg/kg) administered IV every 3 days for a total of 12 injections Sertraline: 2 mg/kg by gavage daily (0.2 mL), starting on day 0 and continued throughout the experiment	Sertraline inhibits P‐glycoprotein in drug‐resistant ovarian cancer cells (NAR), blocking drug efflux Enhances doxorubicin cytotoxicity in resistant cells, more effectively than fluoxetine or verapamil Increases intracellular doxorubicin retention in NAR cells; no effect in sensitive OVCAR‐8 cells In vivo, sertraline + doxorubicin slows tumor growth and extends survival in NAR xenograft mice Combination with DOXIL further improves tumor regression and survival compared to DOXIL alone Effective at low, clinically relevant doses (2 mg/kg/day), supporting potential for clinical use
Kuwahara et al. (2015), Japan [31]	Investigate the antitumor effects of SSRIs and SNRIs on HCCs	*Cancer type*: Liver *Cell lines used*: HepG2 *Animal model*: None	Cell viability Caspase‐3/7 assay	*IC*_*50*_: 1.24 ± 0.0551 μM (0.425 ± 0.0189 μg/mL)	IC_50_ ranking (lowest to highest): Sertraline > paroxetine > duloxetine > fluvoxamine > escitalopram > milnacipran Sertraline (2 μM) significantly increased caspase‐3/7 activity Among the tested agents, HepG2 cells were most sensitive to sertraline Caspase pathway activation is involved in sertraline's antitumor effects in HepG2 cells
Hallett et al. (2016), Canada [32]	Identify small molecules that target BTIC in a transgenic mouse model of breast cancer Determine whether SSRIs, including sertraline, could reduce BTIC activity, inhibit tumor formation, and enhance the effects of chemotherapy	*Cancer type*: Breast *Cell lines used*: MMTV‐Neu (N202) transgenic strain *Animal model*: Female FVB/N mice	High‐throughput screening (Alamar Blue assay) Sphere‐forming assay Secondary sphere‐forming assay Kaplan–Meier survival analysis Histology TUNEL assay IHC Synergy analysis (Bliss model)	*IC*_*50*_: Inhibition of sphere formation = 2.3–2.5 μM across three independent mouse tumor sphere cultures Complete inhibition of sphere formation occurred at 8–9 μM *Animal model*: Mouse mammary tumor cells, with or without sertraline pre‐treatment (5.0 or 7.5 μM) Tumor growth monitored for 15 weeks Mice with ~700 mm^3^ tumors were treated with: ○ Vehicle ○ Sertraline (60 mg/kg IP) ○ Docetaxel (10 mg/kg IP) ○ Sertraline + docetaxel	Sertraline reduced BTIC activity in mouse mammary tumors, as evidenced by its ability to inhibit sphere formation in vitro Pre‐exposure of tumor cells to sertraline before transplantation resulted in delayed tumor formation and reduced tumor incidence Mice transplanted with sertraline‐treated tumor cells (7.5 μM) had significantly fewer tumors, and the average tumor volume was 10‐fold lower compared to controls Sertraline synergized with docetaxel to further inhibit tumor growth, reduce tumor cell proliferation, and increase apoptosis
Schmidt et al. (2016), Sweden [33]	Investigate the synergistic anticancer effects of sertraline and pterostilbene in glioblastoma cells Assess how sertraline potentiates the anticancer effects of pterostilbene	*Cancer type*: Brain *Cell lines used*: U3017MG U3037MG U3047MG U3065MG 41 tumor‐derived glioblastoma cultures from patient samples *Animal model*: None	Cell viability assay Trans well cell migration assay Glioma sphere formation assay Flow cytometry RNA sequencing Western blotting Gene knockdown experiments	*IC*_*50*_: Not reported	Pterostilbene + sertraline + gefitinib synergistically reduced glioblastoma cell viability, migration, and glioma sphere formation Strong synergy observed in EGFR‐amplified glioblastomas, particularly with PIK3CA/PTEN mutations Sertraline induced S‐phase accumulation but unexpectedly activated MAPK signaling (pMEK, pERK), suggesting compensatory feedback RNF11 identified as a potential biomarker for sertraline sensitivity, linked to its ubiquitin‐editing function
Schrödter et al. (2016), Germany [34]	Identify SLC6A3 (dopamine transporter) as a potential biomarker for renal cell carcinoma Investigate SLC6A3 expression levels in renal cell carcinoma tissues and its association with clinical outcomes Assess whether sertraline, an SLC6A3 inhibitor, induces cytotoxic effects in renal cell carcinoma cells	*Cancer type*: Renal *Cell lines used*: A498 Caki‐1 *Animal model*: None	Microarray analysis Real‐time PCR Western blotting IHC Cell viability assay Sertraline dose–response experiment	*IC*_*50*_: Not reported	Sertraline induced dose‐dependent cytotoxicity in Caki‐1 and A498 cells, significantly reducing proliferation at 25–50 μM SLC6A3 identified as a potential biomarker for clear cell renal cell carcinomas, with upregulated mRNA but lower protein levels, suggesting post‐transcriptional regulation High SLC6A3 expression correlated with shorter recurrence‐free survival, indicating prognostic value Sertraline, an SLC6A3 inhibitor, showed anticancer potential, supporting further investigation into its therapeutic efficacy and mechanisms
Boia‐Ferreira et al. (2017), Brazil [35]	To evaluate sertraline as a potential treatment for melanoma by targeting TCTP	*Cancer type*: Melanoma *Cell lines used*: MeWo (p53 wild‐type) A2058 (p53 mutant) B16‐F1 (low metastatic potential) B16‐F10 (high metastatic potential) *Animal model*: C57BL/6 mice with subcutaneous B16‐F10 melanoma xenografts	CellTiter‐Glo luminescence viability Scratch assay Trans well migration Clonogenic assay (soft agar colony formation) Western blotting IHC In vivo tumor growth assessment	*IC*_*50*_: Not reported *Animal model*: Sertraline 10 mg/kg IP for 12 days compared to DTIC (60 mg/kg IP) Tumor growth inhibition: ○ Sertraline alone (10 mg/kg): 84.4% inhibition ○ Sertraline + DTIC: 88% inhibition ○ DTIC alone (60 mg/kg): 47% inhibition	Sertraline reduced melanoma cell viability, colony formation, and migration in vitro Significantly downregulated TCTP expression, leading to increased P53 levels Induced apoptosis in melanoma cells (increased Caspase‐3, decreased Ki67) Inhibited tumor growth more effectively than DTIC in a murine melanoma model Findings suggest sertraline may be repurposed as an anti‐melanoma therapy targeting TCTP
Gwynne et al. (2017), Canada [36]	Investigate the effect of sertraline on breast tumor‐initiating cells (BTIC) and assess its potential as an anticancer agent Assess its impact on sphere‐forming tumor cells, its ability to reduce tumorigenicity, and whether it synergizes with conventional chemotherapy	*Cancer type*: Breast *Cell lines used*: HCC1954 MCF‐7 ZR751 MDA‐MB‐157 MDA‐MB‐453 BT474 BT20 MDA‐MB‐361 T47D BT549 *Animal model*: Female NOD/SCID (Non‐Obese Diabetic/Severe Combined Immunodeficiency)	Sphere‐forming assay Secondary sphere‐forming assay Western blot IF IHC Xenograft tumor formation assay Kaplan–Meier survival analysis Histology/TUNEL	*IC*_*50*_: HCC1954: 1.1 μM MCF‐7: 2.4 μM ZR751: 1.1 μM MDA‐MB‐157: 2.4 μM MDA‐MB‐453: 1.9 μM BT474: 2.7 μM BT20: 3.2 μM MDA‐MB‐361: 1.4 μM T47D: 2.6 μM BT549: 1.3 μM *Animal model*: HCC1954 tumor cells pre‐exposed to sertraline (2.5 or 5 μM) ex vivo Tumor formation monitored over 15 weeks	Sertraline reduced breast tumor cell sphere formation in vitro, indicating BTIC inhibition Primary sphere‐forming assay exposure to sertraline led to lasting cytotoxic effects on BTICs In vivo, sertraline‐treated tumor cells showed delayed tumor formation and reduced incidence in NOD/SCID mice Pre‐treatment with 5 μM sertraline resulted in only 1 out of 10 mice developing tumors after 6 weeks, compared to all 10 in the control group Sertraline effectively reduced BTIC frequency and tumorigenic potential
Li et al. (2017), China [37]	Determine whether inactivation of TCTP by sertraline could sensitize MCF‐7 cells to DNA‐damaging agents Investigate the mechanism by which sertraline suppresses cell resistance to DNA damage Examine the impact of sertraline on homologous recombination repair efficiency Assess the potential of sertraline as a TCTP‐targeting sensitizer for cancer treatment	*Cancer type*: Breast *Cell lines used*: MCF‐7 *Animal model*: None	Immunoprecipitation and LC–MS/MS analysis Cloning and plasmid construction Site‐directed mutagenesis Affinity purification of fusion protein IF Homologous recombination repair assay MTT assay Colony formation assay Flow cytometry Protein half‐life measurement	*IC*_*50*_: Not reported	Sertraline (2.5 μM) inhibited TCTP expression in MCF‐7 cells without affecting morphology or proliferation Increased apoptosis when combined with UVC, etoposide, or olaparib Enhanced cytotoxicity of etoposide and olaparib Clonogenic survival decreased: ○ Etoposide + sertraline: 35.5% down to 13.1% ○ Olaparib + sertraline: ~2× increase in sensitivity Inhibited TCTP disrupted Rad51 stability, impairing homologous recombination repair Increased γH2AX foci, indicating elevated DNA double‐strand breaks
Xia et al. (2017), China [38]	To investigate the antitumor effects of sertraline in acute myeloid leukemia cells, focusing on its ability to induce apoptosis and autophagy, and to explore the interplay between these two pathways in both acute myeloid leukemia cell lines and primary patient‐derived acute myeloid leukemia cells	*Cancer type*: Acute myeloid leukemia *Cell lines used*: NB4 NB4‐R1 NB4‐R2 Primary acute myeloid leukemia cells isolated from the bone marrow of 14 patients *Animal model*: None	Cell cycle analysis Characterization of cell apoptosis Western blotting TEM	*IC*_*50*_: NB4 ○ 22 μmol/L (24‐h treatment) ○ 16.7 μmol/L (48‐h treatment) NB4‐R1 ○ 12.3 μmol/L (24‐h treatment) ○ 10.7 μmol/L (48‐h treatment) NB4‐R2 ○ 22.2 μmol/L (24‐h treatment) ○ 14.2 μmol/L (48‐h treatment)	Sertraline inhibited proliferation of NB4, NB4‐R1 and NB4‐R2 cell lines in a time‐ and dose‐dependent manner Induced cell cycle arrest at G0/G1 or G2/M phase Triggered apoptosis, evidenced by Annexin V staining and caspase‐3/PARP cleavage Induced autophagy, shown by LC3‐II accumulation and autophagic vacuoles Blocking autophagy reduced sertraline‐induced apoptosis and growth inhibition Apoptosis blockade (with Z‐VAD) did not inhibit autophagy or growth suppression Reduced viability and induced apoptosis/autophagy in primary acute myeloid leukemia cells from 14 patients Slightly reduced TCTP expression, suggesting a possible mechanism
Di Rosso et al. (2018), Argentina [39]	Examine how chronic stress promotes tumor growth and metastasis Determine whether stress‐induced tumor progression is mediated through immune suppression or direct effects on tumor cells Assess whether SSRIs can mitigate stress‐related tumor progression by restoring immune function	*Cancer type*: Lymphoma *Cell lines used*: EL4 *Animal model*: Inbred female H‐2b mice, 2–3 months old	QRT‐PCR Trypan blue exclusion test Spontaneous metastasis assay Experimental metastasis assay Trans well migration assay Natural killer cell activity assay Cytotoxic T lymphocyte activity assay Adoptive lymphoid cell transfer experiment Immune cell depletion by complement‐mediated lysis Flow cytometry	*IC*_*50*_: Not reported *Animal model*: Fluoxetine 15 mg/kg/day orally in drinking water Sertraline 20 mg/kg/day orally in drinking water	Chronic stress increased tumor growth and tumor cell dissemination in a mouse model of lymphoma Adoptive immune cell transfer experiments showed that the immune suppression in stressed mice contributed significantly to tumor progression Treatment with fluoxetine or sertraline inhibited the tumor‐promoting effects of chronic stress SSRIs restored normal immune function, improving NK cell activity and cytotoxic responses SSRIs prevented stress‐induced upregulation of MMPs and downregulation of TIMPs, thereby reducing tumor invasiveness
Jiang et al. (2018), China [40]	To investigate sertraline's potential as an anti‐non‐small‐cell lung cancer agent, its synergy with erlotinib, and its autophagy‐inducing mechanism via AMPK/mTOR signaling. To evaluate the combination's efficacy in orthotopic non‐small‐cell lung cancer mouse models	*Cancer type*: Lung *Cell lines used*: A549 H522 H1975 PC9 *Animal model*: 6–8‐week‐old male BALB/cA nude mice 1 × 10^6^ A549‐luc2 cells injected into right lung	Cell viability assay Apoptosis and cell cycle analysis 3‐D colony formation assay Fluorescence analysis of EGFP‐LC3 and mRFP‐EGFP‐LC3 expression TEM Western blotting Gene knockdown A bioluminescent orthotopic NSCLC mouse model	*IC*_*50*_: PC9 = 4.40 μM A549 = 11.10 μM H522 = 10.50 μM H1975 = 9.40 μM PC9/R (erlotinib‐resistant) = 9.60 μM *Animal model*: Mice groups: ○ Vehicle (PBS, daily) ○ Sertraline (50 mg/kg, daily) ○ Erlotinib (50 mg/kg, daily) ○ Sertraline + erlotinib (50 mg/kg each, daily) Median survival (days): ○ Vehicle: 19 ○ Sertraline: 31 ○ Erlotinib: 27 ○ Combination: 40	Sertraline inhibited non‐small‐cell lung cancer cell viability and synergized with erlotinib Promoted autophagic flux, indicated by LC3‐II accumulation and autolysosome formation Reciprocally regulated AMPK/mTOR signaling in non‐small‐cell lung cancer cells AMPK blockade reduced efficacy of sertraline alone or in combination Autophagy inhibition or ATG5/Beclin 1 knockdown decreased effectiveness In orthotopic non‐small‐cell lung cancer mouse models, the combination suppressed tumor growth and prolonged survival Findings support sertraline as a repurposed anticancer agent for non‐small cell lung cancer
Ozunal et al. (2019), Turkey [41]	Investigate the effects of sertraline and sorafenib on HCC Assess whether sertraline enhances the antiproliferative effects of sorafenib Explore apoptosis induction and morphological changes in HepG2 cells treated with sertraline and sorafenib	*Cancer type*: Liver *Cell lines used*: HepG2 *Animal model*: None	XTT cell viability assay Phase contrast microscopy IF microscopy Flow cytometry H&E staining	*IC*_*50*_: 9.6 ± 2.2 μM	Sertraline inhibited HepG2 proliferation in a dose‐dependent manner Sertraline + sorafenib showed synergy, reducing the required sorafenib dose Flow cytometry confirmed enhanced apoptosis with the combination Morphological changes included reduced cell density, vacuolation, and cell destruction Sertraline's effects were serotonin transporter‐independent in HCC cells Combination therapy may target both HCC and comorbid depression
Wang et al. (2019), China [42]	Investigate whether CDC7 inhibition can induce senescence in TP53‐mutant HCC cells Identify and exploit vulnerabilities in senescent liver cancer cells to enhance therapeutic efficacy. Assess the effect of combining CDC7 inhibition with mTOR inhibition or sertraline to promote senolysis and tumor suppression	*Cancer type*: Liver *Cell lines used*: Hep3B Huh7 HepG2 SNU182 SNU398 SNU449 Huh6 SK‐Hep1 PLC/PRF/5 MHCC97H HCCLM3 *Animal model*: 6‐week‐old male BALB/c nude mice Subcutaneously injected with 5 × 10^6^ Huh7 or MHCC97H liver cancer cells into the right posterior flank	CRISPR‐Cas9 genetic screening Clonogenic survival assay SA‐β‐gal staining Western blotting (CDC7, mTOR, apoptosis markers) Caspase‐3/7 apoptosis assay Gene set enrichment analysis Flow cytometry IF RNA sequencing mTOR signaling assays Neutral comet assay Live imaging of mitotic duration Tumor volume measurement in xenografts IHC MRI‐based tumor volume tracking	*IC*_*50*_: Not reported *Animal model*: Mice treated daily via oral gavage for 12–22 days with: ○ XL413 (50–100 mg/kg) to induce tumor senescence ○ AZD8055 (10–20 mg/kg) as mTOR inhibitor ○ Sertraline as a senolytic to selectively kill senescent cells post‐CDC7 inhibition Treatment continued until tumors reached ~2000 mm^3^ or symptoms appeared	CDC7 inhibition selectively induced senescence in TP53‐mutant HCC cells, creating a therapeutic vulnerability A drug screen identified sertraline as a senolytic, inducing apoptosis in senescent HCC cells Sertraline acted by suppressing mTOR signaling and preventing feedback activation In vitro, the combination of CDC7 inhibition and sertraline significantly reduced HCC cell viability In vivo, this combination suppressed tumor growth and prolonged survival in xenograft models Sertraline's senolytic activity was comparable to mTOR inhibitors like AZD8055 Supports a “one‐two punch” strategy: CDC7 inhibition induces senescence, sertraline eliminates senescent cells
Chinnapaka et al. (2020), United States of America [43]	To determine whether the TCTP inhibitor sertraline could target prostate cancer stem cells. To investigate the effects of sertraline on in vitro tumorigenesis and metastasis properties while also delineating its anticancer mechanism	*Cancer type*: Prostate *Cell lines used*: Celprogen (prostate cancer stem cells) PC3 DU145 LNCaP *Animal model*: None	Cell viability Colony formation Spheroid assay Angiogenesis assay Wound healing assay Trans well migration Apoptosis Cell cycle analysis ROS MitoSox staining Hydrogen peroxide assay Thiobarbituric acid reactive species assay Glutathione assay Confocal microscopy Flow cytometry Western blot	*IC*_*50*_: Celprogen = 25 μM PC3, DU145 & LNCaP = 10 μM at 48 h	Sertraline inhibited prostate cancer stem cell viability more potently than dihydroartemisinic Cytotoxicity blocked by NAC, GSH, catalase which confirms oxidative stress‐dependent killing Increased ROS, mitochondrial superoxide, H_2_O_2_; induced lipid peroxidation and depleted GSH Reduced labile iron pool, potentially amplifying ROS toxicity Triggered mitochondrial dysfunction and apoptosis (increased cleaved caspase‐3, cleaved PARP‐1, pH2A.X) Induced G0 cell cycle arrest and downregulated cdc2, phospho‐Histone H3 Activated autophagy (increased LC3‐II, Beclin1, ATG5) Downregulated TCTP (total and phospho), surviving, XIAP, cIAP1 Suppressed stemness by decreasing ALDH1, CD44 and EMT markers (TCF8/ZEB1, LEF1) Disrupted F‐Actin cytoskeleton, impairing migration/invasion; inhibited colony, spheroid, and tube formation
Kharkar et al. (2020), India [44]	Identify existing FDA‐approved drugs that exhibit cytotoxic effects against triple‐negative breast cancer cells, specifically MDA‐MB‐231, and evaluate their potential for drug repurposing as alternative breast cancer treatments	*Cancer type*: Breast *Cell lines used*: MDA‐MB‐231 *Animal model*: None	MTT assay	*IC*_*50*_: Not reported	Of the 70 drugs tested, 11 drugs demonstrated potent cytotoxicity (> 90% inhibition at 10 μM). Sertraline was among the top‐performing hits, showing 98.07% inhibition of MDA‐MB‐231 cells at 10 μM Literature review confirmed sertraline's prior anticancer activity, particularly in colorectal cancer, with IC_50_ values ranging from 8 to 15 μM
Lei et al. (2020), China [45]	Investigate Ser/ICG@Lip, a nanoliposome containing sertraline hydrochloride and indocyanine green (ICG), for dual‐modality imaging and chemo‐photothermal combination therapy against metastatic clear cell renal cell carcinoma	*Cancer type*: Kidney *Cell lines used*: Caki‐1 *Animal model*: None	Cell viability assay (CCK‐8) Near‐infrared fluorescence imaging Photoacoustic imaging Confocal laser scanning microscopy Chemo‐photothermal combination therapy evaluation	*IC*_*50*_: Not reported	Ser/ICG@Lip nanoliposomes successfully synthesized using film‐dispersion and hydration‐sonication methods Encapsulation improved sertraline's stability and reduced its cytotoxicity Dual‐modality imaging (NIR & PAI) confirmed successful uptake and tumor‐targeting potential Chemo‐photothermal therapy with Ser/ICG@Lip was more effective than single chemotherapy (sertraline alone) or photothermal therapy (ICG + laser) Significant tumor cell death observed in the combination therapy group, with strong red fluorescence indicating apoptosis Sertraline may exert direct anticancer effects in metastatic clear cell renal cell carcinoma by inhibiting SLC6A3 and TCTP, two proteins linked to tumor progression
Zinnah et al. (2020), Republic of Korea [46]	To determine the use of sertraline as a sensitizing agent to TRAIL‐mediated apoptosis in lung cancer cells Investigate the molecular mechanisms underlying the anticancer effects of sertraline in combination with TRAIL	*Cancer type*: Lung *Cell lines used*: A549 HCC15 Calu‐3 *Animal model*: None	Cell viability assay LDH assay Western blotting Immunocytochemistry TEM RNA interference	*IC*_*50*_: Not reported	Sertraline sensitized TRAIL‐resistant lung cancer cells, upregulating DR5 and enhancing apoptosis It inhibited autophagic flux via AMPK suppression, effects mimicked by autophagy inhibitors DR5 silencing blocked the apoptosis, confirming its role Findings suggest sertraline may be repurposed as a TRAIL‐sensitizing agent in lung cancer therapy
Duarte et al. (2021), Portugal [47]	Evaluate the potential for drug repurposing by combining central nervous system drugs and antineoplastic agents for the treatment of colorectal and breast cancer Assess whether these combinations could produce synergistic cytotoxic effects	*Cancer type*: Colorectal Breast *Cell lines used*: HT‐29 MCF‐7 *Animal model*: None	MTT assay SRB assay Chou‐Talalay, Bliss Independence, and Highest Single Agent (HSA) synergy models	*IC*_*50*_: HT‐29 = 2.45 μM MCF‐7 = 2.22 μM	Sertraline alone demonstrated potent cytotoxic effects in both colorectal and breast cancer cell lines, making it a strong candidate for drug repurposing Sertraline +5‐FU in colorectal cancer exhibited synergistic effects, suggesting therapeutic potential for combination chemotherapy Further preclinical and clinical studies are warranted to explore its mechanisms and clinical translation for both colorectal and breast cancer treatments
Geeraerts et al. (2021), Belgium [48]	Investigate sertraline hydrochloride as a repurposed drug for targeting serine/glycine synthesis‐addicted breast cancer Identify the molecular mechanisms of sertraline's anticancer effects Evaluate sertraline's efficacy alone and in combination with mitochondrial inhibitors in vitro and in vivo Assess whether sertraline inhibits SHMT, a key enzyme in serine/glycine metabolism	*Cancer type*: Breast Leukemia *Cell lines used*: MDA‐MB‐468 MDA‐MB‐231 MCF‐7 HCC70 Ba/F3 *Animal model*: Immunodeficient NOD‐SCID/IL2γ−/− (NSG) mice	Proliferation assay (IncuCyte Zoom imaging) Cell viability Flow cytometry Stable isotope tracing Metabolite analysis (GC–MS, LC–MS) In vitro PHGDH and SHMT activity assays Computational docking and microscale thermophoresis Xenograft mouse model	*IC*_*50*_: Not reported *Animal model*: Mice received treatments on days 7, 9, 11, 13, 15, 20 and 24. Therapy was administered via intraperitoneal injections at dosages of 2.5 mg/kg sertraline and/or 40 mg/kg artemether. Control mice were treated with DMSO	Sertraline selectively inhibited proliferation in serine/glycine synthesis‐addicted cancer cells (MDA‐MB‐468, HCC70, Ba/F3 RPL10 R98S) Minimal effects were observed in cells relying on extracellular serine/glycine uptake (MDA‐MB‐231, MCF‐7, Ba/F3 WT) Sertraline inhibited SHMT1/2, reducing serine‐to‐glycine conversion Sertraline caused G1‐S phase arrest in MDA‐MB‐468 cells Combination with artemether (antimalarial) enhanced sertraline's effect, inhibiting MDA‐MB‐468 proliferation and tumor growth Xenograft experiments showed significant tumor reduction in MDA‐MB‐468‐derived tumors, but not in MDA‐MB‐231 tumors
Mu et al. (2021), China [49]	To investigate the potential of sertraline in sensitizing drug‐resistant gastric cancer cells to chemotherapy To synthesize and evaluate derivatives of sertraline to improve its anticancer activity To explore the mechanism of action of sertraline and its derivatives in overcoming drug resistance	*Cancer type*: Gastric *Cell lines used*: SGC‐7901/DDP (cisplatin‐resistant gastric cancer cells) *Animal model*: None	Cell viability assay Flow cytometry Western blot analysis Cell cycle analysis	*IC*_*50*_: Sertraline = 18.73 μM Sertraline derivative (trans‐6d) = 5.20 μM	Sertraline sensitized drug‐resistant gastric cancer cells to chemotherapy Modified sertraline derivatives (trans‐6d) showed improved potency (IC_50_ of 5.20 μM) Apoptosis induction and cell cycle arrest were the primary mechanisms of action Sertraline and its derivatives downregulated the PI3K/Akt/mTOR pathway, contributing to cell death The study supports the potential repurposing of sertraline as a chemosensitizer in drug‐resistant gastric cancer treatment
Stapel et al. (2021), Germany [50]	Investigate whether fluoxetine, sertraline, and citalopram affect proliferation or glucose uptake in breast and ovarian cancer cell lines Assess if these SSRIs contribute to increased recurrence and mortality rates in cancer patients	*Cancer type*: Breast Ovarian *Cell lines used*: MCF‐10A MCF‐7 MDA‐MB‐231 MDA‐MSC‐hyb1 MDA‐MSC‐hyb3 SK‐OV‐3 NIH:OVCAR‐3 SCCOHT‐1 SK‐MSC‐hyb1 *Animal model*: None	Fluoroskan assay MTT assay Cell cycle analysis (18)F‐fluorodeoxyglucose uptake assay	*IC*_*50*_: Not reported	SSRIs showed no significant effect on proliferation in most breast and ovarian cancer cell lines at clinically relevant concentrations (10–1000 nM) Higher doses (≥ 10 μM) of sertraline and fluoxetine reduced viability in some cell lines, while citalopram had minimal effects No significant dose‐ or time‐dependent changes in proliferation across most cell lines Marginal increase in glucose uptake observed in SK‐OV‐3 ovarian cancer cells with fluoxetine and sertraline (1 μM), but not citalopram Findings suggest SSRIs are unlikely to drive cancer progression via proliferation or glucose metabolism modulation at therapeutic levels
Ye et al. (2021), China [51]	To investigate the antitumor effects of sertraline in colon cancer, particularly its impact on MEK/ERK signaling and tumor growth To explore the role of sertraline in colon tumorigenesis using SERT‐knockout mice To evaluate whether combining SERT with trametinib or a tryptophan‐restricted diet enhances antitumor efficacy via inhibition of the MEK/ERK pathway	*Cancer type*: Colon *Cell lines used*: SW480 HCT116 *Animal model*: 4–5‐week‐old female BALB/c nude mice, and SERT‐KO or SERT‐WT mice HCT116 cells (1 × 10^6^) subcutaneously injected into right flank Colon tumorigenesis induced by AOM (10 mg/kg i.p.) + 3 cycles of 1.5% DSS in drinking water	Cell transfection Immunoprecipitation assay IF staining qPCR Cell proliferation assay Colony formation assay Colon cancer tissue microarray IHC LC–MS/MS quantification of tryptophan metabolites	*IC*_*50*_: Not reported *Animal model*: Mice treated daily via oral gavage with: ○ Sertraline (30 mg/kg) ○ Trametinib (2 mg/kg)	Sertraline suppressed tumor growth in both allograft and AOM/DSS‐induced colon cancer mouse models Sertraline inhibited MEK/ERK signaling, leading to reduced proliferation of colon cancer cells Combination of sertraline and trametinib (a MEK inhibitor) showed synergistic antitumor effects Sertraline enhanced antitumor efficacy of a tryptophan‐restricted diet, further suppressing ERK activation SERT knockout (SERT‐KO) mice exhibited resistance to colon tumorigenesis, supporting a role for SERT in tumor promotion SERT expression was elevated in colon tumors, and high SERT levels correlated with poor patient prognosis Mechanistically, sertraline blocked SERT‐mediated tryptophan uptake, limiting intracellular tryptophan and suppressing MEK/ERK pathway activation
Zhang et al. (2021), China [52]	Investigate whether sertraline and fluoxetine inhibit HCC growth in vitro and in vivo Determine if sertraline synergizes with sorafenib, enhancing its cytotoxicity Explore the mechanism of action through the PI3K/AKT/mTOR signaling pathway	*Cancer type*: Liver *Cell lines used*: HepG2 Huh7.5.1 Bel7402 SMMC7721 HL7702 (non‐cancerous) *Animal model*: BALB/c nude mice (xenograft with HepG2 cells) C57BL/6 mice, in DEN/CCl_4_‐induced primary liver cancer models	MTT cell viability assay Combination index analysis (Chou‐Talalay method via CompuSyn) Colony formation assay Western blotting IHC Flow cytometry Xenograft tumor growth assessment DEN/CCl_4_‐induced primary liver cancer model Tumor volume and weight measurements	*IC*_*50*_: Bel7402 = 14.49 μM SMMC7721 = 10.3 μM HepG2 = 4.548 μM Huh7.5.1 = 4.457 μM *Animal model*: Sertraline: 20 mg/kg/day by oral gavage Sorafenib: 20 mg/kg/day (alone or in combination)	Sertraline inhibited HCC cell proliferation and colony formation in vitro Downregulated AKT/mTOR signaling, reducing p‐AKT and p‐mTOR expression Reduced Ki67 expression and tumor growth in xenograft and DEN/CCl_4_‐induced liver cancer models Synergized with sorafenib, enhancing cytotoxicity in HepG2 and Huh7.5.1 cells (CI < 1) Combination treatment induced apoptosis and was well tolerated in normal liver cells and mice
Duarte et al. (2022), Portugal [53]	Assess the cytotoxic and synergistic effects of sertraline in colorectal cancer cells and evaluate its biosafety in non‐tumoral fibroblasts Determine potential mechanisms of action through biomarker expression profiling	*Cancer type*: Colorectal *Cell lines used*: HT‐29 MRC‐5 (non‐cancerous lung fibroblasts) *Animal model*: None	MTT cell viability assay Morphological analysis IHC (PPT1, cleaved PARP, Ki67, NF‐κB p65, P‐gp, MRP2) Selectivity index calculation	*IC*_*50*_: HT‐29 = 2.45 μM MRC‐5 (non‐tumoural) = 5.20 μM	Sertraline as a single agent exhibited moderate anticancer activity in HT‐29 cells Morphological changes observed in MRC‐5 cells at 0.5× IC_50_, though viability was not significantly reduced at this dose IHC revealed reduced PPT1 expression in HT‐29 cells, suggesting a lysosomal/apoptotic mechanism
Khodashahri et al. (2022), Iran [54]	Investigate the cytotoxic effects of sertraline on breast cancer cell viability in vitro	*Cancer type*: Breast *Cell lines used*: MDAMB‐231 *Animal model*: None	MTT assay	*IC*_*50*_: 12.6 μg/mL at 24 h 5.2 μg/mL at 48 h	Sertraline at ≥ 3.12 μg/mL significantly reduced MDA‐MB‐231 cell viability after 24 and 48 h 1.56 μg/mL sertraline had no significant effect on cell viability Findings indicate sertraline exerts dose‐dependent cytotoxicity in breast cancer cells
Khodashahri et al. (2022), Iran [55]	Determine the cytotoxic effects of sertraline on cervical cancer (HeLa) cells	*Cancer type*: Cervical *Cell lines used*: HeLa *Animal model*: None	MTT assay	*IC*_*50*_: 16.5 μg/mL at 24 h 4.3 μg/mL at 48 h	Sertraline exhibited significant cytotoxic effects on cervical cancer cells (HeLa) in vitro Prolonged treatment enhanced sertraline's cytotoxicity against cervical cancer cells
Khodashahri et al. (2022), Iran [56]	Investigate the effects of sertraline on cell viability in ovarian (A2780) cancer cells	*Cancer type*: Ovarian *Cell lines used*: A2780 *Animal model*: None	MTT assay	*IC*_*50*_: 11.6 μg/mL at 24 h 5.8 μg/mL at 48 h	Sertraline (6.25–100 μg/mL) significantly reduced cell viability at 24 and 48 h A2780 cells treated with 3.125 μg/mL sertraline showed: ○ No significant viability change at 24 h Significant viability reduction at 48 h compared to controls
Taler et al. (2022), Israel [57]	To compare the effects of sertraline and citalopram on breast cancer proliferation in vitro and tumor progression in vivo To assess the interaction of stress, antidepressant treatment, and tumor growth in a murine breast cancer model To investigate the effects of sertraline on immune system responses	*Cancer type*: Breast *Cell lines used*: 4T1 murine mammary carcinoma cell line *Animal model*: 10‐week‐old female Balb/c mice Subcutaneous injection of 5 × 10^5^ 4T1 cells into the right posterior flank	Cell viability assays (Neutral Red method) Splenocyte viability assay (Alamar Blue) Tumor volume measurement Chronic mild stress model Ex vivo cytokine analysis (TNF‐α) Behavioral analysis (Open Field test)	*IC*_*50*_: Not reported *Animal model*: 4T1 breast cancer cells inoculated in mice, treated daily with: ○ Sertraline (5 or 10 mg/kg IP) ○ Citalopram (10 mg/kg IP) ○ Control (saline IP) Some mice underwent chronic mild stress for 4–6 weeks pre‐implantation Tumor size and survival monitored for 32 days	Sertraline inhibited 4T1 breast cancer cell proliferation in vitro but paradoxically promoted tumor growth in vivo Citalopram had no significant effect on tumor cell viability or growth Sertraline‐treated mice developed larger tumors and had lower survival, though not statistically significant Sertraline reduced anxiety‐like behavior but did not slow tumor progression Sertraline suppressed splenocyte viability, especially under inflammatory conditions TNF‐α levels were elevated in tumor‐bearing mice, with no SSRI‐related differences
Baldissera et al. (2023), Brazil [58]	Assess the potential of sertraline as a therapeutic agent in breast cancer treatment by examining its ability to inhibit TCTP expression and exert antitumor effects	*Cancer type*: Breast *Cell lines used*: MCF‐7 ○ Luminal A, ER+, PR+, HER2− PMC 42 ○ Normal‐like, ER+, PR+, HER2− SKBR3 ○ PR−, ER−, HER2+ MDA‐MB‐231 ○ Basal‐like, triple negative MDA‐MB‐436 ○ Basal‐like, triple negative, ER−, PR−, HER2− *Animal model*: None	MTT viability Protein quantification of cell extracts Gel electrophoresis (SDS‐PAGE) Western blotting Clonogenicity (soft agar method) Cell migration (scratch method) Chemosensitization assay	*IC*_*50*_: Not reported	Sertraline reduces TCTP protein expression in breast cancer cell lines, particularly in triple‐negative breast cancer Sertraline decreases cell viability in a time‐ and dose‐dependent manner across multiple breast cancer subtypes (0.1, 1, and 5 μM tested) Colony formation was significantly reduced by 37% (1 μM) and 55% (5 μM) Sertraline reduced cell migration, with SKBR3 (HER2+) cells showing a 50% decrease in migration at 5 μM after 48 h Sertraline enhanced chemotherapy response in triple‐negative breast cancer cells (MDA‐MB‐231 and MDA‐MB‐436), increasing cytotoxicity when combined with doxorubicin (15 μM) or cisplatin (10 μM) Doxorubicin (15 μM) + sertraline (1 μM): Viability decreased by 55% (vs. 25% with doxorubicin alone) Cisplatin (10 μM) + sertraline (1 μM): Viability decreased by 64% (vs. 45% with cisplatin alone)
Bin Kanner et al. (2023), Israel [59]	To investigate whether three SSRIs could be repurposed as inhibitors of MRP1 and MRP7 transporters to reverse multidrug resistance in cancer cells	*Cancer type*: Skin Ovarian *Cell lines used*: KB‐3‐1 (drug‐sensitive control) KB/CV60 (MRP1‐overexpressing, multidrug‐resistant) SKOV3 (drug‐sensitive control) SKOV3/MRP7 (MRP7‐overexpressing, multidrug‐resistant) *Animal model*: None	MTT viability Molecular docking Normal mode analysis Molecular mechanics‐generalized born surface area calculations Halogen bond analysis	*IC*_*50*_: Vincristine + sertraline (2.5 μM) ○ KB‐3‐1: 1.96 ± 0.33 nM ○ KB/CV60: 1.74 ± 0.18 nM Vincristine + sertraline (5 μM) ○ KB‐3‐1: 1.74 ± 0.26 nM ○ KB/CV60: 1.97 ± 0.27 nM Doxorubicin + sertraline (2.5 μM) ○ KB‐3‐1: 9.18 ± 1.77 nM ○ KB/CV60: 19.85 ± 2.08 nM Doxorubicin + sertraline (5 μM) ○ KB‐3‐1: 8.19 ± 0.94 nM ○ KB/CV60: 12.50 ± 1.53 nM Cisplatin + sertraline (2.5 μM) ○ KB‐3‐1: 1672 ± 249.4 nM ○ KB/CV60: 1755 ± 198.5 nM Cisplatin + sertraline (5 μM) ○ KB‐3‐1: 1852 ± 242.9 nM ○ KB/CV60: 1794 ± 172.9 nM Paclitaxel + sertraline (1 μM) ○ SKOV3: 8.55 ± 0.824 nM ○ SKOV3/MRP7: 19.5 ± 2.16 nM Paclitaxel + sertraline (3 μM) ○ SKOV3: 8.57 ± 0.853 nM ○ SKOV3/MRP7: 13.6 ± 2.13 nM Cisplatin + sertraline (1 μM) ○ SKOV3: 1656 ± 183 nM ○ SKOV3/MRP7: 1793 ± 193 nM Cisplatin + sertraline (3 μM) ○ SKOV3: 1784 ± 175 nM ○ SKOV3/MRP7: 1786 ± 181 nM	Sertraline reversed multidrug resistance in cancer cells overexpressing MRP1 and MRP7 transporters Significantly reduced IC_50_ values of vincristine, doxorubicin, and paclitaxel, restoring drug sensitivity Inhibited MRP1/MRP7 activity via direct interaction with transporter binding pockets Potential for repurposing as a chemosensitizer in resistant cancers
Heylen et al. (2023), Belgium [60]	Determine the therapeutic vulnerabilities of NKX2‐1‐driven cancers Test whether NKX2‐1 expressing cancer cells are more sensitive to serine/glycine synthesis inhibition using repurposed drugs such as sertraline Evaluate the effectiveness of chemotherapy (etoposide) in treating NKX2‐1 driven cancers	*Cancer type*: Leukemia Lung *Cell lines used*: A549 RPMI‐8402 Ba/F3 NCI‐H125 DND41 *Animal model*: 6–8‐week‐old NKX2‐1 tumor bearing NSG mice Tail vein injection of 10^6^ XB41 T‐ALL patient cells	Cell culture Viral vectors CRISPR‐Cas9 genome editing ChlP‐qPCR Motif enrichment qPCR Immunoblotting C tracer analysis Lipidomics Methylation sequencing Incucyte Flow cytometry Xenografts in NOD‐SCID/Il2y (NSG mice) T‐ALL patient samples and patient‐derived xenografts IF Microscope image acquisition and image processing	*IC*_*50*_: Not reported *Animal model*: Mice were randomized and treated for 4 weeks post‐engraftment Sertraline (15 mg/kg IP) was administered twice weekly	NKX2‐1‐expressing cancer cells were highly sensitive to sertraline‐mediated serine/glycine inhibition and etoposide chemotherapy At end‐stage disease, sertraline reduced progression, evidenced by smaller spleens and lower bone marrow infiltration vs. controls A serine/glycine‐free diet + sertraline further enhanced disease suppression
Matsushima‐Nishiwaki et al. (2023), Japan [61]	Investigate whether duloxetine affects TGF‐α‐induced migration of HCC cells Determine the role of SSRIs and SNRIs in cancer cell migration Examine whether the c‐Jun N‐terminal kinase (JNK) signaling pathway is involved in this process	*Cancer type*: Liver *Cell lines used*: HuH7 *Animal model*: None	Transwell cell migration assay Western blot Pharmacological inhibition assays	*IC*_*50*_: Not reported	Sertraline increased HuH7 HCC cell migration in response to TGF‐α, similar to duloxetine and fluvoxamine (SSRIs), but not reboxetine (NRI), suggesting a serotonin‐specific effect Sertraline activated JNK phosphorylation without affecting p38 MAPK or AKT JNK inhibition (SP600125) blocked migration, confirming JNK's role Findings suggest sertraline promotes HCC cell migration via serotonin‐mediated JNK activation, raising concerns about potential cancer progression
Fatehi et al. (2024), Iran [62]	To investigate the effect of sertraline and its combination with agonists and antagonists of RAS (A779, Ang 1–7 and losartan) on viability of MCF‐7 cells along with their effect on apoptosis and cell cycle disruption	*Cancer type*: Breast *Cell lines used*: MCF‐7 *Animal model*: None	Cell viability ROS MTT assay Apoptosis assay	*IC*_*50*_: Not reported	Sertraline, losartan and Ang 1–7 significantly decreased cell viability, induced apoptosis and cell cycle arrest A779 blunted the effect of sertraline on cell viability, ROS generation and cell cycle arrest Combination treatment of sertraline with losartan as well as Ang 1–7 caused a remarkable decline in cell viability
Fayyaz et al. (2024), Pakistan [63]	Evaluate sertraline's cytotoxic effects, focusing on apoptosis induction and cell cycle arrest in breast cancer cells Investigate sertraline as a potential treatment for HER2+ breast cancer	*Cancer type*: Breast *Cell lines used*: AU565 BT‐474 MCF‐7 BJ (human fibroblasts) *Animal model*: None	MTT Annexin V‐FITC/PI staining assay DAPI staining Caspase‐3/7 activity Cell cycle analysis RT‐PCR	*IC*_*50*_: AU565 = 13.8 ± 1.1 μM BT‐474 = 17.54 ± 1.63 μM MCF‐7 = 14.6 ± 0.57 μM BJ (human fibroblast cells) = 16.4 ± 0.4 μM	Sertraline exhibited significant anticancer activity against HER2+ breast cancer cells through apoptosis induction and G2/M cell cycle arrest Cytotoxicity in normal fibroblasts suggests potential side effects, highlighting the need for further mechanistic studies and in vivo validation before clinical application
Gouveia et al. (2024), Portugal [64]	Explore the repurposing of CNS drugs for the treatment of squamous cell carcinoma of the bladder Evaluate the cytotoxic potential of these CNS drugs and compare their efficacy with conventional antineoplastic agents (5‐FU, gemcitabine, imatinib)	*Cancer type*: Bladder *Cell lines used*: UM‐UC‐5 *Animal model*: None	Cell viability assay Cytotoxicity assay Cell morphology visualization	*IC*_*50*_: Sertraline = 1.9 μM Paroxetine = 8.45 μM Chlorpromazine = 5.03 μM 5‐FU = 4.10 μM Gemcitabine = 0.0116 μM Imatinib = > 10 μM	Sertraline exhibited the highest cytotoxic activity against UM‐UC‐5 cells among the tested CNS drugs Sertraline significantly reduced cell viability in a concentration‐dependent manner Morphological analysis revealed that sertraline‐treated cells became rounded and granulated, indicative of potential apoptosis induction Sertraline could be a promising candidate for repurposing in bladder cancer therapy
Gul et al. (2024), Turkey [65]	To evaluate the cytotoxic effects of sertraline and capecitabine, alone and in combination, on breast cancer cell lines and to assess their potential as a repurposed treatment strategy	*Cancer type*: Breast *Cell lines used*: MDA‐MB‐231 MCF‐7 MCF10A *Animal model*: None	Cell viability assay Trypan blue Neutral red assay Investigation of caspase‐3‐8 and 9 enzyme amounts DNA fragmentation Investigation of mTOR amount Molecular docking CompuSyn combination analysis	*IC*_*50*_: MCF‐7 = 51.3554 ng/mL (24 h) MDA‐MB‐231 = 16.86 ng/mL (24 h), 247.937 ng/mL (48 h) MCF10A = 362.91 ng/mL (48 h)	Sertraline/capecitabine combination decreased cell viability compared to drug use alone No significant difference in caspase‐3, ‐8, ‐9, and DNA fragmentation in cancer cells Reduction in mTOR levels observed Suggested death mechanism may be autophagy
He et al. (2024), China [66]	Investigate the anticancer effect of sertraline on colorectal cancer	*Cancer type*: Colorectal *Cell lines used*: HCT116 SW480 MC38 CT26 *Animal model*: 6‐week‐old female BALB/c mice CT26 cells (5 × 10^5^) subcutaneously injected into the right flank	Cell viability assay Western blot analysis Immunocytochemistry staining mCherry‐GFP‐LC3B plasmid transfection	*IC*_*50*_: CT26 = 5.76 μM (48 h) MC38 = 7.25 μM (48 h) HCT116 = 12.12 μM (48 h) SW480 = 10.10 μM (48 h) *Animal model*: After 4 days, mice were randomized into two groups (*n* = 5): ○ Vehicle (0.9% saline) IG ○ Sertraline (50 mg/kg/day) IG Tumor volume (mm^3^) (0.5 × length × width^2^) and body weight were measured every 2 days	Sertraline showed potent anticancer effects against colorectal cancer in vitro and in vivo Sertraline inhibited Akt‐ and STAT3‐driven proliferation but did not affect apoptosis, pyroptosis, ferroptosis, or mitophagy Sertraline induced autophagosome accumulation while blocking autophagic flux in colorectal cancer cells
He et al. (2024), China [67]	To investigate sertraline and paclitaxel's antitumor effects in the treatment of colorectal cancer, alone or in combination	*Cancer type*: Colorectal *Cell lines used*: CT26 MC38 *Animal model*: None	MTT assay Western blotting Combination index analysis using CalcuSyn software	*IC*_*50*_: MC38 = 10.53 μM CT26 = 7.47 μM	Sertraline exerted effective anticancer activity in colorectal cancer cells and sertraline synergistically enhanced the efficacy of the chemotherapeutic agent paclitaxel against colorectal cancer Sertraline augmented paclitaxel‐induced autophagy by increasing autophagosome formation indicated by elevated LC3‐II/I ratio and promoting autophagic flux by degrading autophagy cargo receptor SQSTM1/p62
Jacob et al. (2024), United States of America [68]	To investigate the anti‐glioblastoma activity of specific antipsychotics and antidepressants in vitro and in vivo	*Cancer type*: Glioblastoma *Cell lines used*: U87‐MG OSU2 ACPK4 *Animal model*: None	Cell viability assay Western blot Lysosomal membrane permeability assay Proximity ligation assay (LAMP1 + cathepsin D co‐localization) Real‐time ATP rate assay LC–MS/MS metabolomics	*IC*_*50*_: Not reported	Sertraline exerted cytotoxic effects in glioblastoma cells by disrupting lysosomal integrity and inhibiting Akt/mTORC1 signaling Lysosomal accumulation of sertraline led to membrane permeability, resulting in cell death PTEN‐intact glioblastomas showed increased sensitivity to sertraline Combination therapy with EGFR or Akt inhibitors enhanced sertraline sensitivity, leading to redox collapse Sertraline‐induced cell death was partially rescued by lysosomal function restoration using bafilomycin A1
Sánchez‐Castillo et al. (2024), Netherlands [69]	Investigate the metabolic adaptations of the serine/glycine pathway in non‐small‐cell lung cancer upon radiotherapy Assess the effectiveness of sertraline, a SHMT inhibitor, in enhancing radiotherapy response Examine systemic metabolic changes and immune response modulation in non‐small‐cell lung cancer patients and preclinical models	*Cancer type*: Lung *Cell lines used*: NCI‐H1299 Calu‐6 NCI‐H520 HCC15 NCI‐H125 NCI‐H1975 Lewis lung carcinoma (LLC) Primary bronchial epithelial cells (PBEC) from healthy donors *Animal model*: C57BL/6 mice injected with 1 × 10^6^ LLC cells in the right flank	Mass spectrometry‐based metabolomics 13C6‐glucose tracing metabolomics Real‐time confluency imaging Clonogenic survival assay Colony replating assay Limiting dilution spheroid assay Cytoplasmic ROS Western blotting Cytokine array Cell cycle analysis γH2AX DNA damage assay Cleaved caspase‐3 apoptosis assay Tumor growth monitoring Metabolite and cytokine analysis in blood plasma Immunoblot analysis	*IC*_*50*_: Not reported *Animal model*: Sertraline (15 mg/kg i.p.) started on day 7 Radiotherapy (15 Gy, LINAC) on day 10 at 200 mm^3^ tumor size Sertraline given every 3 days until euthanasia (day 17) Tumors excised, weighed, and analyzed for metabolites, cytokines, and immune markers	Sertraline targeted serine/glycine metabolism to enhance non‐small cell lung cancer radiotherapy response It inhibited cancer cell recovery and modulated the tumor microenvironment Combination therapy reduced proliferation, clonogenic survival, and self‐renewal in vitro In vivo, sertraline suppressed tumor growth and induced systemic metabolic changes Increased ROS levels led to oxidative stress‐induced cell death Modulated immunity by lowering Galectin‐1 and increasing NK cell granzyme B Enhanced non‐small cell lung cancer radiosensitivity by impairing metabolic recovery and boosting antitumor immunity
Sharaf et al. (2024), India [70]	Evaluate the anticancer effects of a novel drug combination consisting of sertraline, plumbagin, and ormeloxifene in triple‐negative breast cancer Assess apoptosis, tumor progression, angiogenesis, and metastasis following treatment Compare the efficacy of the combination therapy against standard treatments like tamoxifen and doxorubicin	*Cancer type*: Breast *Cell lines used*: MDA‐MB‐231 *Animal model*: None	MTT assay Acridine Orange/Ethidium Bromide nuclear morphology analysis Flow cytometry Caspase‐3 assay Cell cycle analysis Colony formation assay Chorioallantoic membrane assay qRT‐PCR	*IC*_*50*_: Not reported	Sertraline exhibited potent cytotoxicity against triple‐negative breast cancer Reduced viability by 98.07% at 10 μM, comparable to doxorubicin Induced apoptosis and caused cell cycle arrest Suppressed colony formation and angiogenesis Modulated key cancer‐related genes (VEGF, P53, P21, ZEB1, HSP70)
Tsuboi et al. (2024), Japan [71]	To investigate the resistance of tumor‐derived endothelial cells (TDECs) to anti‐angiogenic therapy To identify new anti‐angiogenic drugs that can target glioblastoma, particularly those effective against VEGF‐independent angiogenesis To evaluate the potential of sertraline as an anti‐angiogenic agent in combination with the VEGF receptor inhibitor axitinib	*Cancer type*: Glioblastoma *Cell lines used*: 005 (mouse) ○ Differentiated into tumor‐derived endothelial cells U87ΔEGFR (human) Human umbilical vein endothelial cells Mouse brain microvascular endothelial cells *Animal model*: 6‐week‐old female BALB/c‐nu/nu (nude) mice Transplanted with 005 or U87ΔEGFR cells in the right frontal lobe	Cell culture and differentiation assays (005 cells into TDECs) Tube formation assay WST‐1 cell proliferation assay RNA sequencing Western blotting Magnetic resonance imaging IHC Kaplan–Meier survival analysis	*IC*_*50*_: Sertraline ○ TDEC = 4.64 μM ○ MBMECs = No significant effect Axitinib ○ TDECs = No significant effect ○ MBMECs = 13.68 μM ○ HUVECs = 5.45 μM *Animal model*: Sertraline: ○ 25 mg/kg SC, daily, from Day 14 (005 model) Axitinib: ○ 25 mg/kg IP, every other day, from Day 14 (005 model)/Day 5 (U87ΔEGFR model) Anti‐VEGF‐A Antibody (LEAF): ○ 2.5 mg/kg IP, twice weekly, from Day 14 (005 model) MRI and IHC analysis conducted 2–4 weeks after treatment	TDECs were resistant to VEGF‐targeting therapy, which contributed to anti‐angiogenic resistance in glioblastoma Sertraline effectively inhibited tube formation in TDECs, while standard VEGF inhibitors (Axitinib, anti‐VEGF antibody) had no impact Sertraline reduced expression of angiogenesis‐related genes (Lama4 and Angpt2), which play a role in non‐VEGF tumor angiogenesis Combination therapy (sertraline + axitinib) significantly prolonged survival in mice compared to either treatment alone Sertraline's anti‐angiogenic effects were VEGF‐independent, making it a promising candidate for overcoming resistance to VEGF inhibitors
Alvarez‐Valadez et al. (2025), France [72]	Investigate whether sertraline and indatraline induce lysosomal cholesterol accumulation and immunogenic cell death in cancer cells Explore the mechanism of lysosomal membrane permeabilization and antitumor immune activation Assess the anticancer efficacy of sertraline and indatraline in vitro and in vivo	*Cancer type*: Bone Lung Head and neck Cervical Breast Skin Fibrosarcoma *Cell lines used*: U2OS A549 CAL27 HeLa MCF7 B16‐F10 MCA205 *Animal model*: Immunocompetent C57BL/6J mice	Filipin and BODIPY‐cholesterol staining Confocal microscopy Flow cytometry ATP and HMGB1 release assays Prophylactic vaccination‐rechallenge assays In vivo tumor growth and survival studies T‐cell depletion experiments Immunoblotting qRT‐PCR molecular docking for NPC1/2 binding TEM	*IC*_*50*_: Not reported *Animal model*: Single‐dose IP treatment (sertraline 10 mg/kg) in mice delayed MCA205 tumor growth in a T‐cell‐dependent manner	Sertraline caused lysosomal cholesterol accumulation in cancer cells by blocking cholesterol binding to NPC1/2 transporters and upregulating PLA2G15 It triggered lysosomal membrane permeabilization, disrupting autophagy and inducing cell death In in vivo models, sertraline‐treated cancer cells served as prophylactic vaccines, inducing tumor‐specific immune memory and protecting mice against rechallenge A single sertraline injection in mice significantly delayed tumor growth via a T‐cell‐dependent immune response
Bagdonaite et al. (2025), Lithuania [73]	Assess lysosomal membrane microviscosity as a cancer biomarker Investigate the effects of CADs (sertraline, astemizole) on lysosomal lipid order in cancer and noncancer cells	*Cancer type*: Lung Brain Breast Liver *Cell lines used*: A549 U‐87 MCF‐7 HepG2 *Animal model*: None	Synthesis of BODIPY‐Lys probe for FLIM imaging FLIM of lysosomal microviscosities ROS imaging with DCFH‐DA Lysosomal localization by co‐staining with neutral red Live‐cell imaging, FLIM analysis, and 3D rendering of lysosomes	*IC*_*50*_: MCF‐7 = 27.5 μM HepG2 = 21 μM	Sertraline increased lysosomal membrane microviscosity in both cancerous and noncancerous cells In cancer cells, this is linked to saturated lipid and ROS‐related lysosomal changes Cancer cells showed heterogeneous lysosomal responses to sertraline, indicating variable drug effects and lipid metabolism Sertraline's ability to alter lysosomal lipid order may underlie its anticancer potential
Bardaweel et al. (2025), Jordan [74]	To explore sertraline's anticancer effects in prostate cancer cell lines To determine its mechanisms of action and potential synergistic effects when combined with conventional chemotherapeutic agents To validate computational predictions of anticancer effects via in vitro assays	*Cancer type*: Prostate *Cell lines used*: PC3 DU‐145 *Animal model*: None	MTT viability assay Drug combination assay (with cisplatin, 5‐FU, raloxifene) Wound healing assay Soft agar colony formation assay Annexin V‐FITC/PI apoptosis assay Cell cycle analysis (flow cytometry) Real‐time PCR for genes: Bcl‐2, CASP8, DR5, VEGF	*IC*_*50*_: PC3 ○ 23.31 μM (24 h) ○ 13.47 μM (48 h) ○ 9.39 μM (72 h) Combination Treatment ○ Sertraline (1:1) reduced cisplatin IC_50_ from 16.57 to 7.91 μM ○ Sertraline (1:1) reduced 5‐FU IC_50_ from 19.10 to 7.33 μM ○ Sertraline (2:1) reduced raloxifene IC_50_ from 20.51 to 11.51 μM DU‐145: ○ 24.51 μM (24 h) ○ 24.34 μM (48 h) ○ 16.31 μM (72 h) Combination Treatment ○ Sertraline (1:2) reduced cisplatin IC_50_ from 12.63 to 2.98 μM ○ Sertraline (5:1) reduced 5‐FU IC_50_ from 119.10 to 35.53 μM ○ Sertraline (3:1) reduced raloxifene IC_50_ from 60.54 to 14.94 μM	Sertraline significantly reduced cell viability in PC3 and DU‐145 prostate cancer cells (IC_50_≈9–24 μM) Inhibited cell migration Reduced colony formation (anchorage‐independent growth) Induced apoptosis (Annexin V/PI assay) Caused G0/G1 cell cycle arrest. Downregulated Bcl‐2 and VEGF (pro‐survival and angiogenesis genes) Upregulated caspase‐8 and DR5 (extrinsic apoptosis pathway) Showed synergistic/additive effects with cisplatin, 5‐FU, and raloxifene in both cell lines

Abbreviations: 3‐MA, 3‐methyladenine; Akt, protein kinase B (commonly known as Akt); AMPK, AMP‐activated protein kinase; BTIC, breast tumor‐initiating cell; DMSO, dimethyl sulfoxide; DR5, death receptor 5; DSS, dextran sodium sulfate; DTIC, dacarbazine; EMT, epithelial‐mesenchymal transition; ER, endoplasmic reticulum; FBS, fetal bovine serum; GC–MS, gas chromatography–mass spectrometry; HCC, hepatocellular carcinoma; IC_50_, half maximal inhibitory concentration; IF, immunofluorescence; IG, intragastric; IHC, immunohistochemistry; IP, intraperitoneal; JNK, c‐Jun N‐terminal kinase; LC–MS, liquid chromatography‐mass spectrometry; LC3, microtubule‐associated protein 1A/1B‐light chain 3; LDH, lactate dehydrogenase; LD_50_, median lethal dose; mTOR, mechanistic target of rapamycin; NK, natural killer; P‐gp, P‐glycoprotein; PHGDH, phosphoglycerate dehydrogenase; PI3K, phosphoinositide 3‐kinase; qPCR, quantitative polymerase chain reaction; ROS, reactive oxygen species; SERT, serotonin transporter; SHMT, serine hydroxymethyltransferase; SRB, sulforhodamine B; SSRIs, selective serotonin reuptake inhibitors; T‐ALL, T‐cell acute lymphoblastic leukemia; TCTP, translationally controlled tumor protein; TEM, transmission electron microscopy; TIMP, tissue inhibitor of metalloproteinases; TNF‐α, tumor necrosis factor alpha; TRAIL, tumor necrosis factor‐related apoptosis‐inducing ligand; VEGF, vascular endothelial growth factor.

**TABLE 2:** Population‐based studies on the anticancer effects of sertraline.

Author/year/country	Research aims	Study design/cohort	Data analysis	Key findings
Steingart et al. (2003), Canada [75]	To assess whether antidepressant (AD) use increases breast cancer risk To evaluate risk by class (SSRIs, TCAs, MAOIs) and timing/duration of use	*Design*: Population‐based case–control study (Ontario Cancer Registry, June 1996–May 1998) *Cases*: 3133 women aged 25–74 years with primary breast cancer *Controls*: 3062 women without breast cancer, matched by age and residence	Multivariate logistic regression ○ Adjusted for potential confounders including age, height, BMI, reproductive factors, family history, alcohol use, hormone therapy, psychiatric history Exposure variables analyzed: ○ Ever/never AD use ○ Duration of AD use ○ Age at first/last use ○ Menopausal status at use ○ Individual AD types (e.g., fluoxetine, paroxetine, sertraline)	Sertraline use was associated with a modestly increased breast cancer risk Age‐adjusted odds ratio (AOR): 1.58 (95% CI: 1.03–2.41) Multivariate‐adjusted odds ratio: 1.45 (95% CI: 0.88–2.40); not statistically significant No clear association with duration, timing, or recency of use The elevated risk for SSRIs overall was also statistically significant in age‐adjusted models (AOR: 1.33; 95% CI: 1.07–1.66), but not in fully adjusted analyses Potential mechanisms include elevated prolactin and altered estrogen or carcinogen metabolism via cytochrome P450 pathways
Ashbury et al. (2012), Canada [76]	To evaluate whether use of SSRIs is associated with an increased risk of breast cancer, using degree of serotonin reuptake inhibition as a proxy for prolactin‐elevating potential	*Design*: Population‐based case–control study using linked Canadian health databases (2003–2007) *Cases*: 2129 women with primary invasive breast cancer *Controls*: 21 297 age‐matched controls	Logistic regression Stratified analysis: ○ Examined risk separately for premenopausal (< 55 years) and postmenopausal (≥ 55 years) women Sensitivity analysis: Repeated analyses by modifying the reference group from “no prescriptions” to “≤ 1 prescription” to account for potential misclassification	No increased risk of breast cancer with: ○ High inhibitor SSRI use: OR: 1.01 (95% CI: 0.88–1.17) ○ Lower inhibitor SSRI use: OR: 0.91 (95% CI: 0.67–1.25) ○ Duration of use (≥ 24 prescriptions vs. 1–23 prescriptions) was also not associated with increased risk A possible signal was noted for long‐term sertraline use (≥ 24 prescriptions): ○ OR: 1.83 (95% CI: 0.99–3.40), borderline significant, based on only 12 cases ○ No effect modification by menopausal status (age < 55 vs. ≥ 55 years)
Ma et al. (2025), China [77]	To investigate the association between antidepressant use and cancer morbidity and mortality To evaluate these associations for different antidepressant classes (SSRIs, TCAs, SNRIs) and site‐specific cancers	*Design*: Prospective cohort study using the UK Biobank (2006–2010) with 13.6 years median follow‐up *Cases*: 421 529 participants initially free from cancer and cardiovascular disease Self‐reported antidepressant use at baseline *Controls*: 1:1 propensity score matching on 20 covariates, resulting in 26 372 matched pairs (users vs. non‐users)	Cox proportional hazards regression to estimate HRs Adjusted for sociodemographic, lifestyle, and clinical confounders Stratified analyses by demographics and health factors (e.g., smoking, alcohol use, comorbidities) Bonferroni correction for multiple comparisons (*α* = 0.002 for site‐specific cancers)	Antidepressant use was associated with a lower risk of cancer morbidity (HR 0.89) and mortality (HR 0.91) overall SSRIs (notably fluoxetine, citalopram, sertraline) linked to 17%–23% lower cancer morbidity/mortality, especially colorectal cancer (HR 0.75) and malignant melanoma (HR 0.67) Sertraline use specifically associated with a 21% lower risk of overall cancer morbidity In contrast, TCAs (e.g., amitriptyline) associated with an increased cancer‐related mortality (HR 1.19) and site‐specific increases (e.g., mesothelioma, lung cancer) Findings were robust in stratified and sensitivity analyses, suggesting potential protective effects of SSRIs in cancer prevention but warranting cautious interpretation due to residual confounding and healthy user bias
Mørch et al. (2017), Denmark [78]	To determine whether antidepressant use is associated with epithelial ovarian cancer risk To compare the effects of different antidepressant classes (SSRIs, TCAs, others) on cancer risk To assess the influence of specific antidepressants	*Design*: Nested case–control study using Danish national registries *Cases*: 58 706 age‐matched controls (aged 30–84 years) *Controls*: 15 540 women without breast cancer (10:1 matching ratio)	Conditional logistic regression Sensitivity analyses, incorporated mixed use of antidepressants in the analyses for the individual antidepressant classes Analyses stratified according to antidepressant class, individual drug use and by the duration and intensity of antidepressant use	SSRIs were associated with a reduced risk of epithelial ovarian cancer (OR: 0.85; 95% CI: 0.74–0.96) Citalopram, paroxetine, and sertraline were linked to a lower ovarian cancer risk, with citalopram showing the strongest association (OR: 0.78; 95% CI: 0.66–0.93) Other antidepressants (tricyclic antidepressants‐TCAs and others) showed no significant association with ovarian cancer risk The findings suggest potential chemopreventive properties of SSRIs, possibly due to their ability to induce apoptosis in ovarian cancer cells
Chan et al. (2018), Taiwan [79]	To investigate whether SSRI use, including sertraline, is associated with risk of HCC	*Design*: Case–control study using Taiwan's National Health Insurance Research Database (1999–2013) *Cases*: 59 859 HCC patients *Controls*: 285 124 age‐ and sex‐matched controls	Conditional logistic regression Healthy user bias adjustment Magnesium oxide was used as a negative control exposure to test for potential bias	Sertraline use was associated with a lower risk of HCC in a dose‐dependent manner: ○ 1–28 DDD: aOR 0.84 (95% CI: 0.73–0.95) ○ 29–365 DDD: aOR 0.82 (95% CI: 0.71–0.94) ○ ≥ 366 DDD: aOR 0.54 (95% CI: 0.40–0.72) All SSRIs showed a similar protective effect against HCC Findings support a potential chemopreventive role of SSRIs, including sertraline, in HCC
Li et al. (2024), Taiwan [80]	To evaluate whether psychotropic drug use, including sertraline, affects breast cancer risk in women with bipolar disorder or major depressive disorder To assess whether cumulative sertraline exposure (cDDD) influences this risk	*Design*: Nested case–control study using Taiwan's National Health Insurance Research Database *Cases*: 1564 women developed breast cancer *Controls*: 15 540 women without breast cancer (10:1 matching ratio)	Multivariable logistic regression adjusting for: ○ Age, diagnosis type (bipolar disorder/major depressive disorder), medical and psychiatric comorbidities, CCI score, healthcare usage, socioeconomic factors OR and 95% CI calculated to estimate breast cancer risk by exposure group	Long‐term use (cDDD ≥ 365) was significantly associated with reduced breast cancer risk: ○ OR: 0.77, 95% CI: 0.61–0.97, *p* = 0.029 Moderate use (cDDD 30–179) also showed a protective effect: ○ OR: 0.68, 95% CI: 0.55–0.82, *p* < 0.001 No increased risk was observed at any dosage level of sertraline Sertraline may exert anti‐inflammatory effects, contributing to reduced carcinogenic risk Compared to SSRIs like paroxetine or fluoxetine, sertraline is a weaker CYP2D6 inhibitor, posing less interference with tamoxifen metabolism, which is relevant in breast cancer treatment
Busby et al. (2018), United Kingdom [81]	To identify existing medications that may increase or reduce breast cancer risk by combining connectivity mapping (a bioinformatics technique) with pharmacoepidemiologic analysis of United Kingdom primary care data	*Design*: Nested case–control study using the UK Clinical Practice Research Datalink linked to the National Cancer Data Repository *Cases*: 45 147 breast cancer cases *Controls*: 45 147 matched controls (matched on age, year, and GP practice)	Conditional logistic regression Sensitivity analyses adjusting for prescription patterns, missing data, and alternative case definitions	Sertraline use was not associated with breast cancer risk (OR: 1.01, 95% CI: 0.92–1.11) No significant effect even with long‐term use (> 365 DDDs) (OR: 0.98, 95% CI: 0.84–1.14) Bendroflumethiazide showed a small increase in risk (OR: 1.11, 95% CI: 1.06–1.15), despite predictions suggesting a protective effect
Wernli et al. (2009), United States of America [82]	To evaluate the association between AD use and risk of breast cancer To assess risks by antidepressant class, specific drugs, and by age and BMI	*Design*: Population‐based case–control study conducted in Wisconsin (2003–2006) *Cases*: 2908 women aged 20–69 years with newly diagnosed invasive breast cancer, identified via the Wisconsin Cancer Reporting System *Controls*: 2927 age‐matched women randomly selected from licensed drivers with no breast cancer diagnosis	Logistic regression Models adjusted for age, year of interview, parity, age at first live birth, BMI, family history of breast cancer, menopausal status, age at menopause, mammography use, and hormone therapy Stratified analyses conducted by antidepressant class, individual drugs, duration, age, and BMI	Sertraline use was not associated with an increased or decreased risk of breast cancer Odds ratio for ever use of sertraline: 0.98 (95% CI: 0.73–1.33) No associations observed for former or current use, or by duration of use Findings remained null across BMI subgroups and after adjusting for confounders Supports the broader conclusion that SSRIs, including sertraline, are not linked to elevated breast cancer risk
Pocha et al. (2013), United States of America [83]	To assess whether SSRI use is linked to HCC risk in hepatitis C patients To explore serotonin‐related mechanisms and SSRI effects on cancer development	*Design*: Retrospective cohort study using the US Veterans Affairs Clinical Case Registry (2000–2009) *Population*: 109 736 patients with chronic hepatitis C infection *Exposure group*: 36 192 had filled ≥ 1 SSRI prescription	Multivariable Cox regression Adjustments made for age, gender, cirrhosis, hepatitis B, HIV, alcohol use, obesity, liver disease history, psychiatric conditions, and medication use Time‐dependent and subgroup analyses included assessment by duration of follow‐up and presence of cirrhosis	No significant increase in the risk of HCC associated with SSRI use: HR = 0.96 (95% CI, 0.87–1.05) Sertraline was the second most commonly prescribed SSRI, used by 22% of SSRI‐exposed patients No evidence of a dose‐dependent increase in HCC risk with higher SSRI exposure No interaction found between SSRI use and cirrhosis status in relation to cancer risk These findings support the safety of SSRIs, including sertraline, with respect to HCC risk in patients with hepatitis C virus infection

Abbreviations: AD, antidepressant; AOR, age‐adjusted odds ratio; BMI, body mass index; CCI, Charlson Comorbidity Index; cDDD, cumulative defined daily dose; CI, confidence interval; CYP2D6, cytochrome P450 2D6; DDD, defined daily dose; EGFR, epidermal growth factor receptor; GP, general practitioner; HCC, hepatocellular carcinoma; HIV, human immunodeficiency virus; HR, hazard ratio; HRT, hormone replacement therapy; MAOI, monoamine oxidase inhibitor; OR, odds ratio; SSRI, selective serotonin reuptake inhibitor; TCA, tricyclic antidepressant; VA, veterans affairs.

**TABLE 3:** Summary of studies integrating population‐level data with in vitro and in vivo experiments.

Author/year/country	Research aims	Experimental methods	Population study design	Key findings
Christensen et al. (2016), United States of America [84]	To investigate the effects of SSRIs on ovarian cancer progression and survival; to assess the influence of 5‐HT and sertraline on ovarian cancer cell proliferation and 5‐HT receptor signaling	*Cancer type*: Ovarian *Cell lines used*: SKOV3 CP20 HEYA8 2774 ES2 TOV112D OV90 SW626 UWB1.289 CaOV3 *Animal model*: Female athymic nude mice (8–12 weeks old) Daily IP injections of 5‐HT (1 or 10 mg/kg) or sertraline (10 mg/kg)	*Design*: Multicenter retrospective cohort study (1994–2010) *Population*: 773 women with histologically confirmed primary invasive epithelial ovarian, fallopian tube, or peritoneal cancer *Exposure group*: Antidepressants used: SSRIs (16%), SNRIs (6%), TCAs (3%), others (NaSSAs, NDRIs, SARIs)	*Experimental*: Sertraline and 5‐HT significantly increased proliferation in several ovarian cancer cell lines, notably SKOV3, CP20, and ES2. Most lines overexpressed 5‐HT2A mRNA (up to 1600‐fold) Sertraline increased Ki67 expression in vitro and in vivo; in mice, tumor weight doubled (not significant, *p* = 0.16), but Ki67 staining significantly increased (*p* < 0.001). No effect on CD31 or caspase‐3 expression, suggesting the proliferative effect was not angiogenic or anti‐apoptotic *Population*: SSRI use was significantly associated with shorter PFS (HR = 1.3, 95% CI: 1.0–1.6, *p* = 0.03) No significant association with OS (HR = 1.1, 95% CI: 0.9–1.3). Logistic regression suggested a non‐significant trend toward increased progression risk (OR = 1.44, *p* = 0.12) Authors recommend caution with SSRI use in patients with existing ovarian malignancies due to potential tumor‐promoting effects
Sánchez‐Castillo et al. (2024), Netherlands [85]	To investigate whether sertraline disrupts serine/glycine metabolism in glioblastoma and enhances radiotherapy efficacy To assess whether serine metabolism gene expression (e.g., PSPH) predicts prognosis and immunotherapy response in glioma patients	*Cancer type*: Brain *Cell lines used*: U118‐MG GA‐MG LN‐18 GL261 Patient‐derived glioblastoma models (1919 and 2012.2) *Animal model*: Mouse xenografts with patient‐derived GBM cells (1919 and 2012.2) Sertraline (5 mg/kg IP) and Chloroquine (50 mg/kg IP) administered daily	*Design*: Retrospective cohort study of glioma patients diagnosed between 2004 and 2016 *Cases*: 216 glioma patients (median age at diagnosis 60 years [IQR: 49–67])	*Experimental*: Sertraline disrupted serine/glycine metabolism, reducing glioblastoma cell proliferation, migration, and clonogenic survival Mechanisms included PSPH inhibition, NADH/ATP/nucleotide depletion, and ROS accumulation ^13^C_6_‐glucose tracing confirmed blockade of serine biosynthesis Combination with chloroquine enhanced apoptosis and galectin‐1 downregulation, leading to increased NK cell activation and granzyme B production In vivo, sertraline + radiotherapy significantly reduced tumor size and suppressed tumor‐derived serine metabolism and nucleotide biosynthesis *Population*: High glycine levels were significantly associated with poorer survival across glioma subtypes (*p* < 0.001; adjusted HR = 1.20, 95% CI: 1.02–1.41). PSPH overexpression (present in ~25% of GBMs) correlated with increased galectin‐1 expression and immunosuppressive tumor environments GBM patients with high PSPH had worse responses to anti‐PD1 therapy (HR = 4.475, 95% CI: 1.334–15.02, *p* = 0.0104) The study links metabolic reprogramming to treatment resistance and identifies sertraline as a potential sensitizer

Abbreviations: 5‐HT, 5‐hydroxytryptamine (serotonin); ATP, adenosine triphosphate; Ca, calcium; CD31, platelet endothelial cell adhesion molecule‐1; CI, confidence interval; ES2, endometrioid serous carcinoma cell line (ES2); GBM, glioblastoma multiforme; GL261, mouse glioblastoma cell line; HR, hazard ratio; IQR, interquartile range; IP, intraperitoneal; Ki67, proliferation marker protein Ki‐67; mRNA, messenger RNA; NADH, nicotinamide adenine dinucleotide (reduced form); NaSSAs, noradrenergic and specific serotonergic antidepressants; NDRIs, norepinephrine‐dopamine reuptake inhibitors; NK, natural killer; OR, odds ratio; OS, overall survival; PFS, progression‐free survival; PD1, programmed cell death protein 1; PSPH, phosphoserine phosphatase; ROS, reactive oxygen species; SARI, serotonin antagonist and reuptake inhibitor; SSRI, selective serotonin reuptake inhibitor; SNRI, serotonin‐norepinephrine reuptake inhibitor; TCAs, tricyclic antidepressants.

**TABLE 4:** Summary of studies on sertraline treatment across cancer types categorized by country and year of publication.

Author (year)	Country	Cancer types																
		Bladder	Blood	Bone	Brain	Breast	Cervical	Colorectal	Gastric	Head/neck	Kidney	Liver	Lung	Oral	Ovarian	Prostate	Sarcoma	Skin
Di Rosso et al. (2018) [39]	Argentina		✓															
Geeraerts et al. (2021) [48]	Belgium		✓			✓												
Heylen et al. (2023) [60]	Belgium		✓										✓					
Boia‐Ferreira et al. (2017) [35]	Brazil																	✓
Baldissera et al. (2023) [58]	Brazil					✓												
Steingart et al. (2003) [75]	Canada					✓												
Lin et al. (2010) [23]	Canada		✓			✓												
Ashbury et al. (2012) [76]	Canada					✓												
Hallett et al. (2016) [32]	Canada					✓												
Gwynne et al. (2017) [36]	Canada					✓												
Li et al. (2017) [37]	China					✓												
Xia et al. (2017) [38]	China		✓															
Jiang et al. (2018) [40]	China												✓					
Wang et al. (2019) [42]	China											✓						
Lei et al. (2020) [45]	China										✓							
Mu et al. (2021) [49]	China								✓									
Ye et al. (2021) [51]	China							✓										
Zhang et al. (2021) [52]	China											✓						
He et al. (2024) [66]	China							✓										
He et al. (2024) [67]	China							✓										
Ma et al. (2025) [77]^a^	China	✓	✓		✓	✓	✓	✓	✓	✓	✓	✓	✓	✓	✓	✓	✓	✓
Mørch et al. (2017) [78]	Denmark					✓												
Tuynder et al. (2004) [19]	France		✓			✓		✓					✓					✓
Alvarez‐Valadez et al. (2025) [72]	France			✓		✓	✓			✓			✓				✓	✓
Schrödter et al. (2016) [34]	Germany										✓							
Stapel et al. (2021) [50]	Germany					✓									✓			
Kharkar et al. (2020) [44]	India					✓												
Sharaf et al. (2024) [70]	India					✓												
Khodashahri et al. (2022) [54]	Iran					✓												
Khodashahri et al. (2022) [55]	Iran						✓											
Khodashahri et al. (2022) [56]	Iran														✓			
Fatehi et al. (2024) [62]	Iran					✓												
Gil‐Ad et al. (2008) [21]	Israel							✓										
Amit et al. (2009) [22]	Israel		✓															
Tzadok et al. (2010) [24]	Israel				✓													
Drinberg et al. (2014) [30]	Israel														✓			
Taler et al. (2022) [57]	Israel					✓												
Bin Kanner et al. (2023) [59]	Israel														✓			✓
Kuwahara et al. (2015) [31]	Japan											✓						
Matsushima‐Nishiwaki et al. (2023) [61]	Japan											✓						
Tsuboi et al. (2024) [71]	Japan				✓													
Bardaweel et al. (2025) [74]	Jordan															✓		
Bagdonaite et al. (2025) [73]	Lithuania				✓	✓						✓	✓					
Sánchez‐Castillo et al. (2024) [69]	Netherlands												✓					
Sánchez‐Castillo et al. (2024) [85]	Netherlands				✓													
Duarte et al. (2021) [47]	Portugal					✓		✓										
Duarte et al. (2022) [53]	Portugal							✓										
Fayyaz et al. (2024) [63]	Pakistan					✓												
Gouveia et al. (2024) [64]	Portugal	✓																
Zinnah et al. (2020) [46]	Republic of Korea												✓					
Schmidt et al. (2016) [33]	Sweden				✓													
Chien et al. (2011) [25]	Taiwan													✓				
Huang et al. (2011) [26]	Taiwan															✓		
Lin et al. (2013) [27]	Taiwan			✓														
Chan et al. (2018) [79]	Taiwan											✓						
Li et al. (2024) [80]	Taiwan					✓												
Ozunal et al. (2019) [41]	Turkey											✓						
Gul et al. (2024) [65]	Turkey					✓												
Busby et al. (2018) [81]	UK					✓												
Reddy et al. (2006) [20]	USA																	✓
Wernli et al. (2009) [82]	USA					✓												
Pocha et al. (2013) [83]	USA											✓						
Chen et al. (2014) [28]	USA											✓						
Chen et al. (2014) [29]	USA											✓						
Christensen et al. (2016) [84]	USA														✓			
Chinnapaka et al. (2020) [43]	USA															✓		
Jacob et al. (2024) [68]	USA				✓													

*Note:* Green cells denote in vitro studies, pink cells represent animal studies, yellow cells indicate retrospective population studies, and blue cells represent dual studies (in vitro/animal and population).

^a^ Additional cancer types analyzed by Ma et al. (2025) [77] not included in this table: thyroid, mesothelioma, gallbladder/biliary tract, pancreatic, uterine/endometrial, and small intestine cancers.

### 3.3 Mechanisms of Sertraline's Anticancer Activity

**FIGURE 2:**
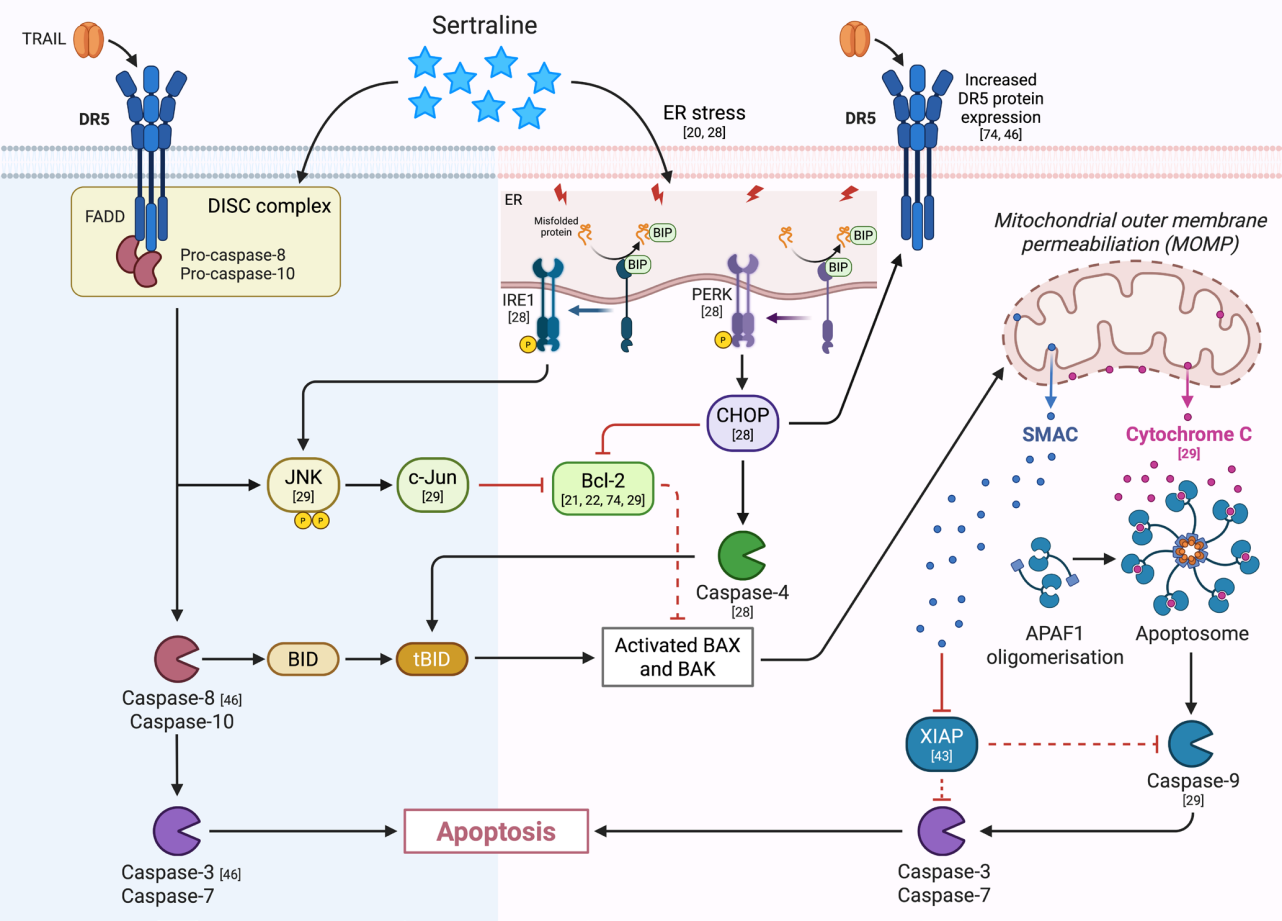
Mechanistic pathways of sertraline‐induced apoptosis via ER stress and death receptor signaling in cancer cells. Illustration created in Biorender.com by authors. Dashed red lines (Bcl‐2, XIAP) indicate inhibitory interactions that normally prevent apoptosis within the cell.

Sertraline exerts anticancer effects through diverse cellular mechanisms. Apoptosis induction is the most consistently reported, involving caspase‐3/7 activation, Bcl‐2 inhibition, endoplasmic reticulum (ER) stress, mitochondrial dysfunction, reactive oxygen species (ROS) generation, and lysosomal membrane permeabilization [20, 22, 26, 43, 68] (Figure 2). Sertraline also disrupts protein synthesis and survival pathways, with mTOR inhibition and impaired translation initiation noted in breast and lymphoma models [23, 27]. A recurrent target is TCTP, where sertraline promotes p53 stabilization and impairs DNA repair via Rad51 destabilization, particularly in breast, prostate, and melanoma cells [35, 37, 43]. Additionally, sertraline interferes with metabolism, inhibiting SHMT1/2, thereby blocking serine/glycine biosynthesis, depleting nucleotides, and promoting oxidative stress [48, 69, 86]. Additional mechanisms include autophagy flux inhibition and altered calcium signaling, suggesting that sertraline engages multiple context‐dependent cytotoxic pathways [25, 66].

### 3.4 Cancer Types and Patterns of Sensitivity to Sertraline

Sertraline demonstrated cytotoxic activity across a wide range of malignancies. Breast cancer was the most studied cancer type, with consistent dose‐dependent reductions in viability observed across hormone receptor‐positive (MCF‐7, BT474), HER2+ (AU565), and triple‐negative cell lines (MDA‐MB‐231, MDA‐MB‐436), with IC_50_ values ranging from 1.1 to 17.5 μM [36, 58, 63]. Notably, sertraline appeared particularly effective in triple‐negative and TCTP‐high subtypes. In colorectal cancer models (e.g., HT‐29, HCT116), sertraline exhibited IC_50_ between 2.4 and 14.7 μM and outperformed traditional chemotherapeutics in multidrug‐resistant cell lines [21, 87]. Sensitivity was also confirmed in glioblastoma, melanoma, prostate, ovarian, liver, lung, and bladder cancers. Selectivity toward cancer cells over normal fibroblasts was reported in several studies, though high‐dose exposure could still exert toxicity, underscoring the need for in vivo evaluation [53, 63].

### 3.5 Effects of Sertraline on Tumor Progression and Survival in Animal Models

Preclinical animal studies reveal that sertraline suppresses tumor progression and, in some cases, prolongs survival, even when used as monotherapy. In melanoma‐bearing mice, sertraline alone achieved up to 84% tumor growth inhibition, outperforming the alkylating agent dacarbazine, and further enhanced efficacy when used in combination [35]. Similar effects were observed in breast, liver, and colorectal models, with reductions in tumor volume and proliferation markers such as Ki67 and increased apoptosis [40, 52].

### 3.6 Interactions Between Sertraline and Standard Cancer Therapies

Sertraline frequently acts as a chemosensitizer, reversing drug resistance and augmenting standard therapies. In cell lines overexpressing efflux pumps such as MRP1, MRP7, or P‐gp, sertraline increased intracellular concentrations of doxorubicin, vincristine, and paclitaxel, thereby lowering their IC_50_ values and restoring drug sensitivity [30, 59]. This transporter inhibition is supported by molecular docking studies that reveal sertraline's affinity for key efflux domains. Synergistic cytotoxicity has also been observed in combinations with DNA‐damaging agents (e.g., etoposide, olaparib), autophagy modulators, and chemotherapy backbones (e.g., paclitaxel, 5‐FU, cisplatin) [37, 47, 67]. In glioblastoma models, combining sertraline with the autophagy inhibitor chloroquine enhanced immune activation and cell death, while in lung cancer, it sensitized tumors to radiotherapy by impairing serine/glycine metabolism and redox balance [85].

### 3.7 Clinical Evidence for Sertraline in Cancer Treatment

Population studies offer a reassuring safety signal for sertraline use in cancer contexts and hint at possible protective associations. Multiple large‐scale cohort and case–control studies from Canada, Taiwan, the US, and Europe found no evidence that sertraline use increased breast cancer risk, even with prolonged exposure [81, 82]. In some cases, long‐term use was associated with reduced incidence, especially in psychiatric populations [80]. These findings counter early concerns regarding prolactin elevation or CYP2D6 inhibition. Beyond breast cancer, sertraline use was associated with significantly reduced risk of hepatocellular carcinoma (HCC) in both general and hepatitis C‐infected populations, with a dose–response effect suggesting potential chemoprevention [79, 83]. A similar protective association was reported for epithelial ovarian cancer in SSRI users [78]. Although these findings do not establish efficacy, they lend support to ongoing exploration of sertraline in oncology. Importantly, they also suggest that repurposing this agent is unlikely to exacerbate cancer risk, a prerequisite for further clinical translation.

### 3.8 Sertraline and the Hallmarks of Cancer

The hallmarks of cancer [88] provide a structured framework for interpreting sertraline's anticancer actions (Table 5). For example, 72.4% of studies report sertraline engaging cell death pathways, making this the most consistently targeted hallmark. Proliferative signaling is also frequently affected, with 56.9% of studies demonstrating suppression of cell growth or viability. Metabolic disruption is prominent in 27.6% of studies, implicating sertraline in the modulation of nutrient sensing and mTOR signaling. Additionally, 20.7% of studies implicate effects on invasion and metastasis, while 13.8% highlight modulation of growth suppressor pathways, and another 13.8% describe replicative immortality. Smaller subsets address genome instability and mutation (12.1%), angiogenesis (10.3%), and phenotypic plasticity (10.3%). Only 5.2% of studies address immune evasion or non‐mutational epigenetic reprogramming. Overall, sertraline's profile is dominated by apoptotic and antiproliferative actions, with emerging evidence for effects on metabolism, tumor adaptability, and resistance. However, key gaps remain in our understanding of its impact on host–tumor interactions and less‐explored hallmarks such as epigenetic regulation.

**TABLE 5:** Categorization of studies based on the hallmarks of cancer, showing the frequency and percentage of studies associated with each hallmark (studies may overlap across multiple hallmarks).

Hallmarks of cancer	References	*n* (%)
Resisting cell death	[20, 21, 22, 23, 24, 26, 28, 29, 30, 32, 33, 35, 36, 37, 38, 39, 40, 41, 42, 43, 46, 47, 48, 49, 51, 52, 53, 54, 55, 56, 58, 59, 62, 63, 64, 65, 66, 67, 68, 70, 71, 85]	42 (72.4)
Sustaining proliferative signaling	[20, 21, 23, 32, 33, 35, 36, 38, 40, 41, 42, 43, 45, 47, 48, 49, 51, 52, 54, 55, 56, 57, 58, 59, 60, 63, 64, 65, 66, 67, 70, 87]	33 (56.9)
Deregulating cellular metabolism	[23, 27, 33, 40, 41, 42, 45, 48, 49, 51, 52, 60, 68, 69]	16 (27.6)
Activating invasion and metastasis	[30, 32, 33, 36, 39, 43, 58, 59, 61, 70, 71, 89]	12 (20.7)
Evading growth suppressors	[21, 22, 23, 33, 35, 42, 48, 58]	8 (13.8)
Enabling replicative immortality	[32, 36, 37, 40, 43, 47, 60, 87]	8 (13.8)
Genome instability and mutation	[21, 24, 27, 37, 40, 59, 85]	7 (12.1)
Inducing or accessing vasculature	[30, 35, 43, 59, 70, 71]	6 (10.3)
Unlocking phenotypic plasticity	[30, 37, 42, 43, 59, 70]	6 (10.3)
Avoiding immune destruction	[39, 57, 85]	3 (5.2)
Non‐mutational epigenetic reprogramming	[33, 60, 87]	3 (5.2)

## 4 Discussion

Sertraline, a widely prescribed SSRI, has emerged as a promising candidate for anticancer drug repurposing. Sertraline's cytotoxicity has been demonstrated across diverse tumor models (Table 4), with current evidence pointing toward a complex, multimodal mechanism of action. Rather than acting through a single dominant pathway, sertraline appears to engage a network of interrelated processes to restore sensitivity to apoptosis [63], modulate autophagy [43, 48], disrupt oncogenic signaling [86], and induce metabolic and proteostatic stress [28, 42]. To our knowledge, this is the first review to systematically synthesize the existing literature on these mechanisms, describing how sertraline may exploit core vulnerabilities in cancer cells and highlighting its potential as a repurposed anticancer treatment.

### 4.1 Restoration of Stress‐Induced Death Pathways: Apoptosis, Autophagy, ER Stress, and Metabolic Collapse

One of the hallmarks of cancer, and a major contributor to treatment resistance, is the ability to evade apoptosis and deregulate cell cycle checkpoints. Many conventional cancer treatments, including chemotherapy and radiotherapy, ultimately rely on triggering regulated cell death pathways [90]. Apoptosis is mediated by two converging pathways: the extrinsic pathway, triggered by death receptor signaling, and the intrinsic pathway, initiated by intracellular stressors such as DNA damage, oxidative stress, and ER stress, culminating in caspase activation, mitochondrial outer membrane permeabilization (MOMP), and irreversible cellular dismantling [90, 91]. Malignant cells evade these mechanisms by rewiring apoptotic signaling and altering checkpoint proteins to gain a survival advantage. This is often achieved through overexpression of anti‐apoptotic proteins like Bcl‐2, Bcl‐xL, and Mcl‐1, suppression or mutation of pro‐apoptotic effectors such as BAX, BAK, or caspases, and loss‐of‐function mutations in TP53 [91, 92]. Additionally, alterations in checkpoint control, such as the loss of cyclin‐dependent kinase (CDK) inhibitors, enable uncontrolled cell cycle progression despite genomic instability, fostering survival under cytotoxic stress and promoting therapeutic resistance, heterogeneity, and relapse [93].

Several studies suggest that sertraline restores apoptotic sensitivity in cancer cells. Fayyaz et al. [63] showed that sertraline reduced the viability of AU565 (HER2‐positive breast cancer) cells by downregulating cyclin D1 and upregulating CDK inhibitors p21 and p27, resulting in G1/S cell cycle arrest and caspase‐3‐mediated apoptosis. This mechanism parallels the rationale behind CDK4/6 inhibitors in breast cancer therapy [94, 95, 96]. Sertraline has also been shown to induce mitochondrial membrane depolarization and caspase‐9 activation, with increased γH2AX expression suggestive of genotoxic stress and impaired DNA repair processes [38, 97]. The effects of sertraline on DNA repair have been further elucidated by Malard et al. [98], who found that sertraline indirectly inhibits Rad51, a DNA repair protein, and modulates TCTP, a protein involved in p53 regulation and survival signaling. While sertraline exhibited only weak direct binding to TCTP, its cellular effects mimicked those observed with direct TCTP inhibition, suggesting that interference with pro‐survival chaperones may contribute to its cytotoxicity.

Sertraline also influences autophagy and stress responses, both of which are critical to tumor survival. Autophagy, a tightly regulated catabolic process, recycles cytoplasmic components under metabolic stress and is frequently used by cancer cells to survive harsh conditions. Even under non‐stressed conditions, many tumor cells maintain elevated basal autophagy, reflecting the inherently stressful metabolic environment of malignancy [99]. However, autophagy may also act as a tumor suppressor in some contexts, as shown in mouse liver models where autophagy deficiency led to p62 accumulation, mitochondrial dysfunction, oxidative stress, DNA damage, and genomic instability [100]. Sertraline modulates autophagy in a bidirectional manner depending on cancer type and context. In prostate cancer stem cells, sertraline induces autophagy via mitochondrial dysfunction, ROS accumulation, and AMPK activation, leading to suppression of mTOR signaling [43]. In contrast, in serine/glycine‐addicted breast cancers and glioblastomas, sertraline disrupts autophagic flux by inhibiting SHMT, leading to accumulation of defective autophagosomes and increased susceptibility to apoptosis [48]. In lung cancer models, sertraline combined with erlotinib enhances autophagy and synergistic cell death [40], whereas in tumor necrosis factor‐related apoptosis‐inducing ligand (TRAIL)‐resistant cells, sertraline blocks autophagy, sensitizing them to death receptor 5 (DR5)‐mediated apoptosis [46]. Sertraline also induces ER stress and activates the unfolded protein response (UPR). While the UPR normally restores ER homeostasis, excessive or prolonged ER stress can tip cells into apoptosis [101]. Chen et al. [28] demonstrated that sertraline activates PERK/CHOP and IRE1‐XBP1 signaling branches of the UPR and engages caspase‐4, triggering ER stress‐mediated apoptosis. This mechanism is particularly relevant in secretory tumors such as HCC. It mirrors the action of bortezomib, a proteasome inhibitor that leverages terminal UPR activation to induce tumor cell death [102]. Thus, sertraline's ability to overwhelm metabolic and proteostatic adaptations positions it as a promising agent to restore stress‐induced death pathways in malignancy.

### 4.2 Inhibition of Tumor‐Initiating and Stem‐Like Cells

Tumor‐initiating cells (TICs), or cancer stem‐like cells, are a subpopulation within tumors responsible for relapse, metastasis, and therapeutic resistance due to their ability to self‐renew and resist conventional therapies. TICs exploit developmental signaling pathways, such as Wnt/β‐catenin and STAT3, to maintain stemness and proliferative potential [103]. Recent studies have highlighted the ability of sertraline to selectively impair TIC function across various cancer types. In prostate cancer, sertraline induces mitochondrial dysfunction and lysosomal stress, disrupting energy homeostasis and promoting autophagy‐mediated death of TICs [43]. This mechanism aligns with that of salinomycin and thioridazine, known TIC‐targeting agents that induce lysosomal membrane permeabilization [104]. In glioblastoma models, sertraline downregulates SOX2 and β‐catenin, critical transcription factors for maintaining neural stemness and therapy resistance [105]. In melanoma and colorectal cancer, sertraline suppresses markers associated with epithelial‐mesenchymal transition (EMT), a process linked to stemness and invasiveness [106]. These findings suggest that sertraline may have an important role in targeting tumor maintenance hierarchies, similar to other investigational therapies such as napabucasin (BBI608), which targets STAT3 to suppress relapse and metastasis [103].

### 4.3 Synergistic Effects With Standard Therapies

Sertraline exhibits promising synergy with conventional cancer treatments by targeting metabolic vulnerabilities, autophagy regulation, and apoptotic resistance pathways. Mu et al. [49] demonstrated that sertraline downregulates mTOR signaling, enhancing chemosensitivity in drug‐resistant gastric cancer, paralleling mechanisms exploited by metformin and phenformin [107]. Rather than activating AMPK to inhibit mTOR, sertraline can disrupt autophagy by suppressing AMPK phosphorylation. Zinnah et al. [46] showed that in TRAIL‐resistant lung cancer cells, this suppression upregulated DR5, enhancing caspase‐8 and caspase‐3 activation. Similarly, in non‐small cell lung cancer, sertraline combined with erlotinib enhanced autophagy through the AMPK/mTOR axis [40]. These effects mirror strategies using hydroxychloroquine with temozolomide, which achieved stable disease rates of 69%–73% in advanced solid tumors [108]. Sertraline also sensitizes tumor cells to apoptosis, analogous to venetoclax, a Bcl‐2 inhibitor used in hematologic malignancies [109]. Boia‐Ferreira et al. [35] similarly demonstrated that sertraline enhances the pro‐apoptotic effects of temozolomide in melanoma. Multi‐drug regimens that simultaneously disrupt overlapping survival pathways are increasingly favored in oncology. Sertraline's inclusion in the CUSP9v3 glioblastoma regimen highlights its potential role in preventing adaptive resistance [110]. In HCC models, sertraline enhanced therapeutic efficacy when combined with CDC7 inhibition by promoting apoptosis in senescent tumor cells through mTOR suppression [42]. Overall, these findings validate sertraline's position within emerging low‐dose, multi‐targeted anticancer strategies.

### 4.4 Reconsidering TCTP as a Therapeutic Target

TCTP has long been considered a compelling oncogenic driver, associated with cell proliferation, survival, and resistance to genotoxic stress [111]. Early studies suggested that sertraline exerted anticancer effects through direct TCTP inhibition. For example, Gil‐Ad et al. [21] reported that sertraline reduced viability in T‐cell acute lymphoblastic leukemia cells, accompanied by findings consistent with TCTP inhibition, namely TCTP destabilization, Bcl‐2 downregulation, and activation of caspase‐3. Additional support for the direct TCTP inhibition hypothesis comes from studies demonstrating that sertraline mimics the cellular effects of known TCTP‐targeting agents, such as dihydroartemisinin, by downregulating TCTP expression and promoting apoptotic signaling cascades [112, 113]. These phenotypic effects support the proposition that sertraline may directly target TCTP, contributing to EMT suppression and apoptotic resistance. However, detailed biophysical analyses by Malard et al. [98] showed that sertraline binding to TCTP was weak and non‐specific, even after phosphorylation at serine 46. This challenges the direct targeting hypothesis and suggests that sertraline's anticancer effects are mediated through alternative mechanisms, including mTOR suppression, ER stress induction, and disruption of neurotransmitter pathways. This repositioning mirrors other repurposed agents such as everolimus and thioridazine, which exert anticancer effects by exploiting downstream vulnerabilities rather than direct oncogene inhibition [114, 115].

### 4.5 Limitations and Future Directions

While the preclinical evidence supporting sertraline's anticancer potential is strong, it is important to note that most data is confined to in vitro research, with fewer in vivo studies to date. Although tumor suppression and survival benefits have been demonstrated in xenograft models, translation into clinical success remains uncertain. The pharmacokinetic profile of sertraline itself also poses challenges. Standard antidepressant doses, which are generally within the range of 50–200 mg per day [116], may not achieve the concentrations required to achieve cytotoxicity. Across the included studies, sertraline dosing was as high as 60 mg/kg in an animal model [32], which would equate to 4200 mg in an average 70 kg person. Supratherapeutic doses may be harmful to patients, potentially increasing the risk of serotonin syndrome [117], gastrointestinal and neurological disturbances [118], and drug‐induced liver injury [119]. Thus, innovative delivery approaches such as nanoparticle formulations, prodrugs, or targeted delivery may be necessary. For example, intravesical treatment may be an option in bladder cancers. Furthermore, although the chemosensitizing properties of sertraline are useful in an oncology setting, they could pose liabilities in non‐cancer contexts. For patients on other medications, particularly those reliant on cytochrome P450 metabolism, there is a theoretical risk of increased toxicity [120]. While not well‐characterized clinically [118], this warrants careful drug–drug interaction assessment in future studies. Additionally, the metabolic vulnerabilities exploited by sertraline may not be universally present given the inherent heterogeneity of cancers. In order to rationally select patients, it will be crucial to identify predictive biomarkers, such as serine/glycine addiction, mTOR hyperactivation, or elevated ER stress signatures. Future research should prioritize combination studies, careful toxicological profiling, and early‐phase clinical trials in cancers against which sertraline has shown the most preclinical promise, such as glioblastoma, HCC, non‐small cell lung cancer, and breast cancer.

### 4.6 Summary

This scoping review provides a systematic mapping of the existing evidence regarding the anticancer potential of sertraline, highlighting its multimodal mechanisms of action across apoptosis, autophagy, ER stress, and metabolic signaling pathways. These preclinical studies suggest that sertraline exploits several core vulnerabilities of cancer cells, including defective apoptotic machinery, metabolic reprogramming, and stress adaptation pathways. This forms the basis of its therapeutic repurposing. Importantly, sertraline appears to exert its cytotoxic effects through a network of interconnected biological processes rather than reliance on a single target, positioning it favorably within the emerging landscape of multi‐targeted anticancer strategies. Although promising, these findings are tempered by limitations in mechanistic clarity, pharmacokinetic feasibility, and tumor heterogeneity. In the context of drug repurposing, early‐phase (phase I) clinical trials for sertraline would benefit from leveraging its established safety profile in psychiatric populations. Notably, repurposing pathways such as the FDA's 505(b)(2) New Drug Application can streamline approval processes for existing compounds with novel oncology indications [121]. Ultimately, moving forward, focused efforts to refine mechanistic understanding, identify predictive biomarkers, and evaluate synergistic combinations in in vivo and clinical settings will be essential to realize sertraline's potential as a novel component of cancer therapy.

### 4.7 Nomenclature of Targets and Ligands

Key protein targets and ligands in this article are hyperlinked to corresponding entries in http://www.guidetopharmacology.org, the common portal for data from the IUPHAR/BPS Guide to PHARMACOLOGY [122], and are permanently archived in the Concise Guide to PHARMACOLOGY 2019/20 [123].

## Author Contributions

The authors confirm that all listed authors meet the requirements for authorship.

## Ethics Statement

The authors have nothing to report.

## Consent

The authors have nothing to report.

## Conflicts of Interest

The authors declare no conflicts of interest.

## Supporting information


**Data S1:** prp270168‐sup‐0001‐Supinfo.docx.

## Data Availability

Data are available upon reasonable request to the corresponding author.
