# Correction to “Repurposing the Antidepressant Sertraline: A Systematic Scoping Review of Its Anticancer Mechanisms”

**DOI:** 10.1002/prp2.70220

**Published:** 2026-02-02

C. B. Blum, M.‐J. Dohrmann, L. McCarthy, M. McMenamin, and L. A. O'Callaghan, “Repurposing the Antidepressant Sertraline: A Systematic Scoping Review of Its Anticancer Mechanisms,” Pharmacology Research & Perspectives 13, no. 5 (2025): e70168, https://doi.org/10.1002/prp2.70168.

In the Discussion section, the reference Malard et al. [98] was cited instead of Li et al. [37].

“The effects of sertraline on DNA repair have been further elucidated by Malard et al. [98], who found that sertraline indirectly inhibits Rad51, a DNA repair protein, and modulates TCTP, a protein involved in p53 regulation and survival signaling.”

This should have read:

“The effects of sertraline on DNA repair have been further elucidated by Li et al. [37], who found that sertraline indirectly inhibits Rad51, a DNA repair protein, and modulates TCTP, a protein involved in p53 regulation and survival signaling.”

The correct reference is:

Li, Y., Sun, H., Zhang, C. et al. Identification of translationally controlled tumor protein in promotion of DNA homologous recombination repair in cancer cells by affinity proteomics. Oncogene 36, 6839–6849 (2017). https://doi.org/10.1038/onc.2017.289.

We apologize for this error.

