# Supplementary material for: Repurposing the Antidepressant Sertraline: A Systematic Scoping Review of Its Anticancer Mechanisms
Source: Pharmacol Res Perspect. 2025 Aug 28;13(5):e70168. doi: 10.1002/prp2.70168 (PMC12392137; doi:10.1002/prp2.70168)
Supplement: Supplementary file 1 — Data S1: prp270168‐sup‐0001‐Supinfo.docx. [file PRP2-13-e70168-s001.docx]

**Supplementary information - search terms**

**PubMed:**

(sertraline[Title/Abstract] OR zoloft[Title/Abstract])

AND

(cancer*[Title/Abstract] OR tumo*[Title/Abstract] OR anticancer*[Title/Abstract] OR antitum*[Title/Abstract] OR cytotoxic*[Title/Abstract] OR apopto*[Title/Abstract] OR "cell death"[Title/Abstract] OR proliferati*[Title/Abstract] OR metastas*[Title/Abstract])

**EMBASE:**

(sertraline:ti,ab OR zoloft:ti,ab)

AND

(cancer*:ti,ab OR tumo*:ti,ab OR anticancer*:ti,ab OR antitum*:ti,ab OR cytotoxic*:ti,ab OR apopto*:ti,ab OR 'cell death':ti,ab OR proliferati*:ti,ab OR metastas*:ti,ab)

**SCOPUS:**

(TITLE-ABS(sertraline) OR TITLE-ABS(zoloft))

AND

(TITLE-ABS(cancer*) OR TITLE-ABS(tumo*) OR TITLE-ABS(anticancer*) OR TITLE-ABS(antitum*) OR TITLE-ABS(cytotoxic*) OR TITLE-ABS(apopto*) OR TITLE-ABS("cell death") OR TITLE-ABS(proliferati*) OR TITLE-ABS(metastas*))

**Web of Science:**

((TI=sertraline OR AB=sertraline) OR (TI=zoloft OR AB=zoloft))

AND

((TI=cancer* OR AB=cancer*) OR (TI=tumo* OR AB=tumo*) OR (TI=anticancer* OR AB=anticancer*) OR (TI=antitum* OR AB=antitum*) OR (TI=cytotoxic* OR AB=cytotoxic*) OR (TI=apopto* OR AB=apopto*) OR (TI="cell death" OR AB="cell death") OR (TI=proliferati* OR AB=proliferati*) OR (TI=metastas* OR AB=metastas*))
